# Acoustic Features of Sustained Phonation for Schizophrenia Classification: A Feasibility Study

**DOI:** 10.3390/s26196195

**Published:** 2026-09-30

**Authors:** Luka Jelić, Kristian Jambrošić, Vinko Lešić, Pavo Orepić

**Affiliations:** 1 University of Zagreb Faculty of Electrical Engineering and Computing, 10000 Zagreb, Croatia; kristian.jambrosic@fer.unizg.hr (K.J.); vinko.lesic@fer.unizg.hr (V.L.); 2 Ericsson Nikola Tesla d.d., 10000 Zagreb, Croatia; 3 Linguistic Research Infrastructure, University of Zurich, 8050 Zurich, Switzerland; pavo.orepic@uzh.ch

**Keywords:** schizophrenia, voice analysis, sustained phonation, acoustic features, machine learning, feature selection, data augmentation

## Abstract

Voice and speech are increasingly studied as indicators of mental health, but the acoustic features of sustained phonation in schizophrenia remain underexplored. This feasibility study examined whether a 500 ms sustained vowel /a/ contains meaningful information for distinguishing patients with schizophrenia from healthy controls. Recordings from 84 participants (41 patients, 43 controls) were analyzed using features from eight acoustic domains. Random Forest, XGBoost, and logistic regression were evaluated under four train–test configurations with data augmentation and participant-grouped cross-validation. Feature importance with Random Forest Gini index, supported by SHAP analysis, produced a compact data-driven 15-feature set (DD15). Random Forest trained with fixed DD15 on original and augmented recordings, and evaluated on original recordings, achieved an AUC of 0.887 ± 0.078, while fold-wise feature selection produced a more conservative 15-feature estimate of 0.837 ± 0.086. Amplitude skewness, harmonic-to-noise ratio, and shimmer measures were the most discriminative features. The conservative DD15 estimate was broadly comparable with the stronger alternative representations while retaining interpretability. Additional sensitivity analyses showed that adjustment for measured recording condition variables reduced, but did not eliminate, internal patient-control discrimination. These findings support the feasibility of sustained-vowel acoustic analysis for schizophrenia classification and emphasize the need for standardized, balanced multi-site validation.

## 1. Introduction

Schizophrenia is a severe psychiatric disorder that affects approximately 23 million people globally [1]. It is characterized by positive symptoms (e.g., hallucinations, delusions), negative symptoms (e.g., diminished emotional expression, avolition, alogia), and cognitive deficits [2]. Its diagnosis relies mainly on clinical interviews and standardized rating scales such as the Positive and Negative Syndrome Scale (PANSS) [3]. Although clinically established, these approaches are time-consuming, require specialized training, and provide limited objective physiological information. This has motivated an increasing interest in measurable, non-invasive biological signals that could facilitate earlier detection or more scalable clinical assessment.

Speech and voice have long been recognized as indicators of neurological and psychiatric conditions [4,5]. In schizophrenia, much of this work has focused on speech patterns, including reduced verbal output, atypical pauses, altered prosody, and disturbances in semantic coherence and thought organization [6,7,8,9]. These speech-level markers are valuable because they capture clinically relevant disturbances in communication, cognition, and symptom expression. However, less attention has been given to the acoustic properties of the voice signal itself, even though such features can be measured from brief, simple vocalizations and may reveal abnormalities in phonation or vocal-tract control, independent of linguistic content [10]. The most consistent findings in this area concern reduced pitch range and prosodic modulation, which are both associated with negative symptoms [11]. However, voice perturbation measures such as jitter, shimmer, and harmonic-to-noise ratio show mixed findings, with some studies reporting both reduced [12] and increased perturbation [13]. Moreover, studies focusing specifically on sustained phonation in schizophrenia are comparatively scarce. Mouratai et al. examined sustained vowels in patients with schizophrenia and healthy controls and reported significant group differences in jitter, shimmer, and harmonic-to-noise ratio, indicating that group-related acoustic information can also be present in vocalizations without linguistic content [14]. Nevertheless, whether such information is sufficient for multivariate classification from a very short sustained-vowel segment remains largely unexplored.

Machine-learning methods have been widely applied for schizophrenia voice classification, using general-purpose acoustic feature sets [15,16,17] and, more recently, representations learned from pretrained speech models [18]. Reported performance nevertheless varies considerably, suggesting that no single robust pipeline has yet been established. Small sample sizes increase the risk of overfitting and unstable feature selection, and questions of data augmentation and feature selection stability remain underexplored. Many studies employ broad general-purpose paralinguistic feature sets such as eGeMAPS [19], which provide standardized descriptors but may not capture the acoustic features most relevant for schizophrenia. In addition, pretrained speech embeddings have been explored less extensively in schizophrenia studies using sustained vowels than handcrafted acoustic features, while systematic comparisons remain limited [18,20]. Most studies also rely on connected speech or interview tasks, which carry rich diagnostic signal but do not distinguish between the phonatory, prosodic, and linguistic contributions [8,9,20]. Reproducibility remains a concern [13,20], and cross-linguistic analyses suggest that models performing well within a single dataset may not generalize reliably across independent cohorts and languages [13,20].

This study adopts a minimalist approach, investigating whether a single 500 ms sustained vowel /a/, which substantially reduces linguistic and connected-speech prosodic variation, contains enough acoustic information to distinguish patients with schizophrenia from healthy controls. The 500 ms segment duration followed the stimulus preparation protocol of the parent self–other voice perception study [21], and was retained unchanged for the present classification analysis. Independently, previous work on the acoustic analysis of sustained phonation has also used 500 ms stable segments for perturbation and noise-related voice measures [22], indicating that several acoustic parameters remain stable over short analysis windows. Studies comparing different segment lengths have also shown that several jitter and shimmer measures can remain stable across durations including 500 ms [23]. Therefore, although 500 ms is not assumed to be the optimal duration for all acoustic measures or for schizophrenia classification, it offers a reasonable standardized interval for testing whether a short sustained vocalization contains useful discriminative information. We therefore frame this work as a feasibility study rather than a validated diagnostic tool.

Within that scope, we:

(1) establish baseline classification performance using three different classifiers and two reference feature sets;
(2) evaluate the effect of conservative data augmentation on classification performance with limited data;
(3) construct a compact, data-driven feature set (DD15) and compare it against literature-based, exhaustive, general-purpose, and pretrained embedding representations;
(4) identify acoustic features that consistently contribute to discrimination across complementary linear and nonlinear analyses; and
(5) assess the sensitivity of the resulting signal to recording site, medication dose, symptom severity, and participant sex.

We additionally report exploratory observations on amplitude skewness, the strongest predictor in our analysis, which to our knowledge has not previously been reported as a discriminative acoustic feature in schizophrenia voice research.

## 2. Materials and Methods

### 2.1. Participants

This study is a continuation of our previous research [21], which involved 86 adult participants: 43 patients with schizophrenia (23 with auditory verbal hallucinations (AVH+) and 20 without AVH) and 43 healthy controls. Two patient recordings were excluded from the present analysis due to technical errors, resulting in a total of 84 participants for this study: 41 patients with schizophrenia (23 AVH+) and 43 healthy controls.

The primary patient inclusion criterion was a verified schizophrenia diagnosis according to the International Classification of Diseases, 10th Revision (ICD-10) [24], established through a structured clinical interview performed by a psychiatrist. The main exclusion criterion was being in an acute schizophrenic episode at the time of study enrollment (e.g., experiencing active hallucinations). Additional exclusion criteria included the presence of comorbid psychiatric disorders, any current or previous substance use disorder, neurological disorders, and serious somatic conditions. Past experience of auditory verbal hallucinations was verified through clinical interviews and patient electronic health records. Symptom severity scores were evaluated by an experienced psychiatrist using the Positive and Negative Syndrome Scale (PANSS).

Healthy control participants were recruited from the general population. Eligibility criteria included the absence of self-reported psychiatric and neurological disorders, as well as a lack of hearing impairments. All control participants completed the Cardiff Anomalous Perceptions Scale (CAPS) [25], a questionnaire measuring unusual perceptual experiences, and Peters et al. Delusion Inventory (PDI) [26], a questionnaire assessing delusion-like or unusual belief experiences.

Detailed participant information can be found in Table 1.

The mean PANSS total score indicated that the patient group was experiencing moderate symptom severity, with higher negative subscale scores than positive subscale scores at the group level. Control participants reported low levels of anomalous perceptual experiences and delusional ideation. These levels of responses are common in non-clinical populations and are not indicative of a psychotic disorder in themselves.

### 2.2. Voice Recording and Preprocessing

The participants’ voices were recorded while vocalizing the phoneme /a/ for the duration of 2 to 5 s. The recordings were made with Huawei CM33 headphones with microphone (Huawei Technologies Co., Ltd., Shenzhen, China), with participants instructed to keep the microphone at a fixed distance of 10 cm from their mouth. The same recording equipment (laptop, microphone) was used for all the participants. Each recording was processed with Audacity^®^ 3.4.2 recording and editing software [27]. Recordings were converted from stereo to mono and peak-normalized to −12 dB. Background noise was then reduced using Audacity’s profile-based Noise Reduction effect. A noise-only segment preceding the vocalization was manually selected to obtain a Noise Profile, after which Noise Reduction was applied to the complete recording using a noise reduction of 6 dB, sensitivity of 6.00, and frequency smoothing of 6 bands. No additional high-pass, low-pass, band-pass, notch, or other cutoff filter was applied. As the final step, a 500 ms voice segment was selected manually from the most stable part of the vocalization (the part with the least variations in sound intensity) and saved as a 44.1 kHz 16-bit mono .wav audio file. The recordings were then resampled to 22.05 kHz for augmentation and feature extraction.

These preprocessing steps were inherited from the parent self–other voice perception study [21], in which the 500 ms segments were prepared as standardized auditory stimuli, and were applied identically to patient and control recordings. Their purpose was to reduce differences in recording level and background noise across participants and recording locations rather than to optimize the present classification analysis. All patients were recorded at one clinical site, in the same testing room, as required by the clinical access arrangements for the patient group, while controls were recorded across seven different sites, in different rooms, offices, or laboratories.

### 2.3. Acoustic Features

Voice acoustic features were extracted from each 500 ms recording using parselmouth, librosa, and SciPy libraries [28,29,30,31] in Python 3.12.3. To define the baseline feature sets against which classification models can be compared, a list of the most consistently used acoustic properties across previous schizophrenia and psychiatric voice research was derived (Table 2).

Rather than adopting any single study set used throughout this literature, we assembled a reference set of 14 features: F0 mean, F1–F5 means, local jitter, local shimmer, HNR mean, and MFCC1–5 means, and named it Literature 14. The purpose of this set is to establish a model baseline on a minimum set of voice acoustic features already known and established in schizophrenia research.

To define a second, larger baseline feature set, a pool of 50 features was derived across eight acoustic domains and named Extended 50: voice quality (jitter, shimmer, HNR), prosody (F0 statistics), formants (F1–F5 means), cepstral descriptors (MFCCs and derivatives), spectral descriptors (entropy, centroid, and related measures), chroma (pitch-class energy bands), time/energy (zero crossing rate—ZCR, root mean square—RMS), and a distributional measure (waveform amplitude skewness). Chroma bands represent pitch-class energy, delta and delta-delta MFCCs are first and second time derivatives, spectral entropy quantifies spectral flatness, and pitch_cv is the coefficient of variation of F0.

Both the Literature 14 and the Extended 50 feature sets thus provided compact and extended reference representations for subsequent model analyses (Section 3.1). The complete Extended 50 feature list and its relationship to the Literature 14 and DD15 are provided in Supplementary Table S2.

### 2.4. Classification Models and Evaluation

Three supervised models were evaluated for classification of patients with schizophrenia versus healthy controls:

- Random Forest [35]: 200 trees, maximum depth = 10.
- Extreme Gradient Boosting (XGBoost) [36]: 200 trees, maximum depth = 5, learning rate = 0.1.
- Logistic Regression: maximum iterations = 1000.

A Support Vector Machine (SVM) [37] model (radial basis function (RBF) kernel, C=1.0, γ=scale, no class weighting) was subsequently used as part of the final feature set exploration.

All hyperparameters were specified a priori and were not tuned on the study data, in order to limit additional model-selection flexibility in this small cohort. The reported configurations should therefore be regarded as fixed model settings rather than optimized configurations. Logistic regression was run with a maximum of 1000 iterations to ensure convergence. All stochastic models used a fixed random seed of 42. Features were standardized using StandardScaler before training logistic regression and the SVM, whereas Random Forest and XGBoost were trained on the original feature values.

Model performance was assessed using stratified 5-fold group cross-validation (StratifiedGroupKFold) with participant-level grouping to prevent data leakage between the training and test sets. Evaluation metrics included area under the receiver operating characteristic (ROC) curve (AUC), accuracy, sensitivity, specificity, and F1 score. AUC was reported as the mean and standard deviation across folds. Accuracy, sensitivity, specificity, and F1 score were reported as fold-averaged means unless otherwise stated.

**Figure 1:**
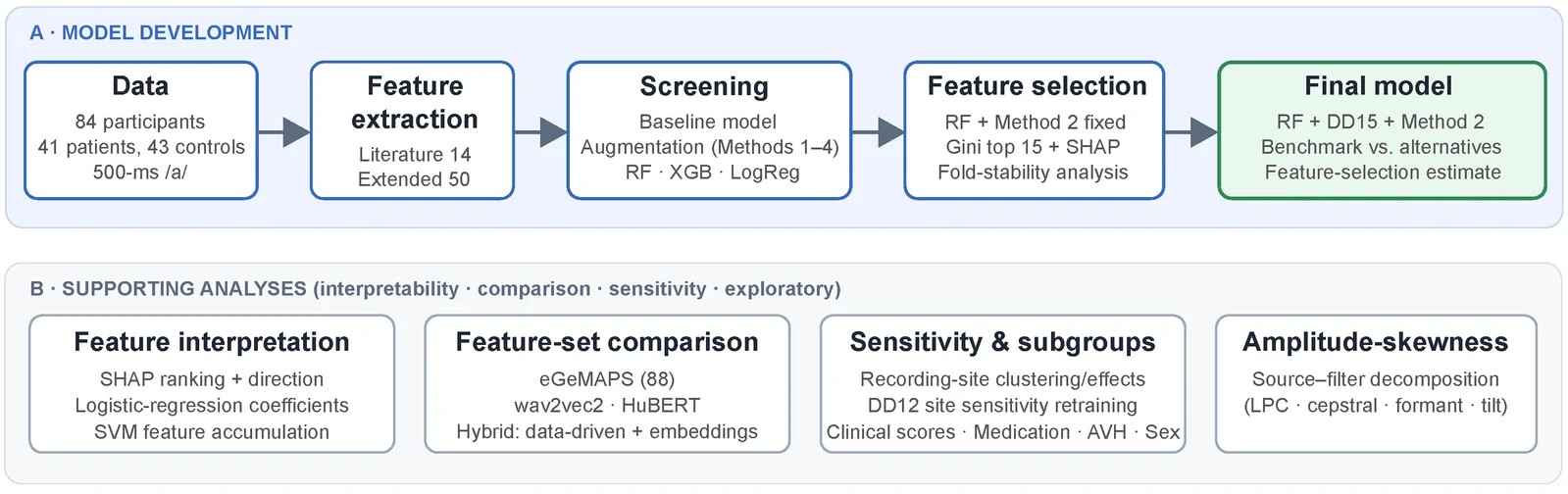
Overview of the analytical workflow. The upper row shows the steps used to develop and evaluate the primary classification pipeline, from feature extraction and augmentation screening to data-driven feature selection and final evaluation. The lower row summarizes the complementary analyses of feature interpretation, feature set comparison, sensitivity, subgroups and exploration. Five-fold cross-validation with fold-wise feature selection repeated feature ranking independently within each training fold and evaluated performance on held-out participants. DD15: Data-driven 15-feature set; RF: Random Forest; XGB: XGBoost; LogReg: logistic regression.

The subsequent analyses consisted of a primary model development pipeline, followed by internal validation and complementary interpretability, sensitivity, subgroup and exploratory analyses (Figure 1).

### 2.5. Data Augmentation

To address the limited sample size, data augmentation was applied to each original recording. Five variants of the original participant’s voice were generated through controlled perturbations in pitch, duration, noise level, and intensity to introduce modest training variation around the original recording:

- Pitch shift: ±0.6% of F0
- Time stretch: ±5% of recording duration
- Additive Gaussian noise: fixed amplitude (σ = 0.005 of full scale)
- Volume change: ±1 dB, approximately ±12% amplitude change

For the Gaussian-noise augmentation, noise was added at a fixed standard deviation (σ=0.005 of full scale) rather than at a fixed target SNR. Consequently, the resulting signal-to-added-noise ratio depended on source level and varied across augmented samples. Across all 269 noise-containing augmented copies in the dataset, the mean SNR was 20.11 ± 4.45 dB (median 19.71 dB; IQR 17.59–21.86 dB; range 11.64–37.40 dB).

**Figure 2:**
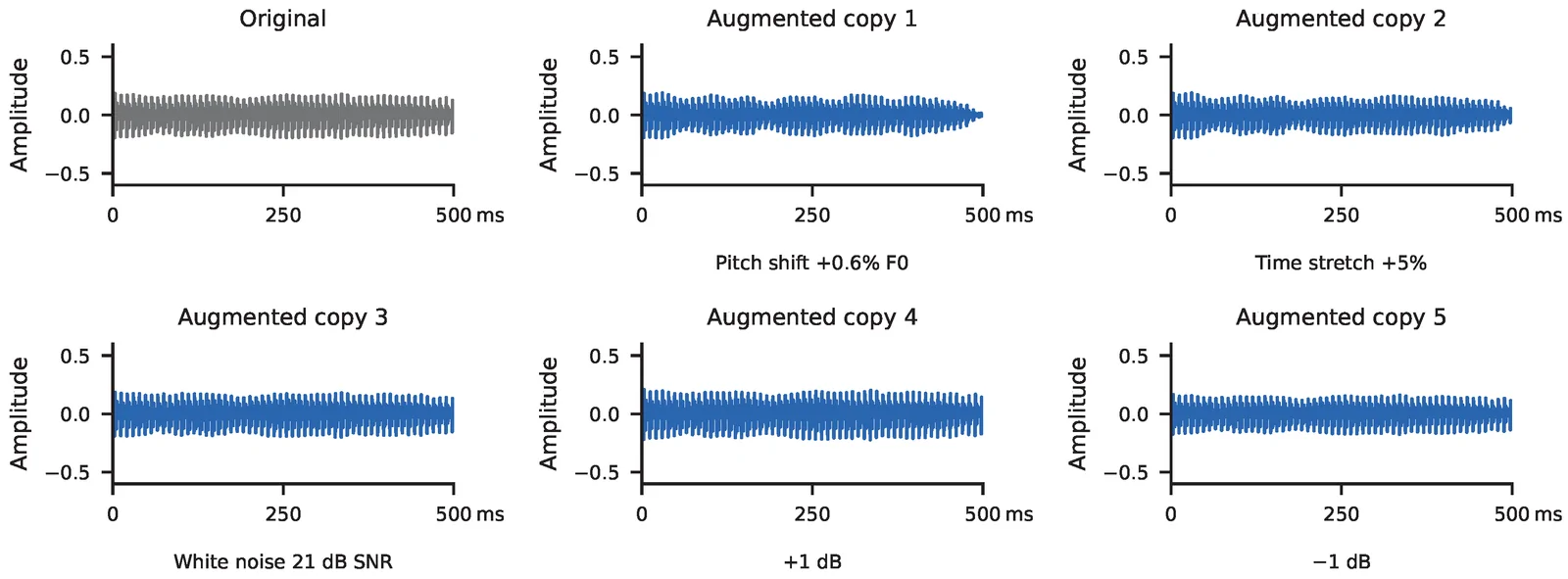
Representative 500 ms voice segment and five augmented copies generated through conservative signal perturbations. Perturbation types are applied individually here for illustration.

Using a random selection of two or three of the above modifications, five augmented copies were generated from each original voice recording, resulting in a total of six voice samples per participant (Figure 2). These variants are synthetic perturbations of a single original recording and should not be interpreted as empirical repeated measurements or as an estimate of true within-participant voice variability.

The aim of data augmentation was to increase the amount of training data while preserving participant-level grouping throughout all analyses. All original and augmented samples from the same participant shared the same group identifier. Therefore, within each cross-validation split, when a participant was held out for testing, neither the original recording nor any of its augmented copies could be used for model training. In the separate fold-wise feature selection analysis, feature ranking was repeated using training fold participants only.

To evaluate its effect on classification performance, four augmentation methods with train–test configurations were defined (Table 3).

These configurations enabled the contribution of data augmentation to be evaluated relative to a baseline condition (Method 1) from three perspectives: (1) whether adding augmented recordings improves generalization to original recordings (Method 2); (2) whether performance gains result from similarity between augmented training and testing recordings (Method 3); and (3) whether training exclusively on augmented recordings is sufficient to achieve generalization to original recordings (Method 4). The corresponding results are reported in Section 3.2.

In the following notation, used throughout the text, the first term denotes the training set and the second term denotes the test set:

- Method 1 (Original/Original)
- Method 2 (Original+Augmented/Original)
- Method 3 (Augmented/Augmented)
- Method 4 (Augmented/Original)

### 2.6. Data-Driven Feature Selection and Fold-Wise Evaluation

*DD15 feature selection.* To identify a compact acoustic representation while maintaining interpretability, a data-driven feature selection procedure was applied to the Extended 50 candidate feature pool. Random Forest under Method 2 (Original+Augmented/Original) was selected for this procedure because it was the only classifier for which adding augmented recordings produced a numerically higher mean AUC while evaluation remained exclusively on original recordings. The procedure consisted of one selection step and two complementary assessments:

- **Gini ranking.** Random Forest Gini feature importance was computed for the Extended 50 feature set under Method 2. Mean Gini importance across the five cross-validation folds was used to rank the candidate features, and the 15 highest-ranked features were retained.
- **Stability analysis.** Selection stability was assessed from the fold-specific top-15 feature lists. Features selected in all five folds were labelled core, those selected in four folds near-core, those selected in three folds variable, and those selected in two folds unstable. Agreement between fold-specific feature sets was summarized using mean pairwise Jaccard similarity, while agreement between feature rankings was assessed using Spearman rank correlation.
- **SHAP comparison and direction.** SHapley Additive exPlanations (SHAP) [38] were computed for the same Random Forest + Method 2 + Extended 50 configuration to provide a complementary importance assessment and characterize the direction of each feature’s contribution toward the patient class prediction.

The 15 features with the highest mean Gini importance were designated as the Data-driven 15-feature set (DD15). The stability and SHAP analyses were used to assess the consistency and direction of the selected features rather than to select additional features (Section 3.3).

*Fold-wise feature selection estimate.* Since DD15 was selected using Random Forest Gini importance on the full dataset, testing the Random Forest directly on DD15 could introduce circularity into the evaluation process, as the same participants were used to both test the model and to select the features. To obtain a more cautious estimate, the analysis was repeated in five rounds using stratified cross-validation grouped by participants. In each round, one group of participants was excluded, while the remaining participants were used to rank the Extended 50 features using Random Forest under Method 2. Separate Random Forest models were then trained using the top eight, ten, twelve, or fifteen features, and tested only on the original recordings of the excluded participants. All recordings from participants who had been excluded, including augmented copies, were omitted from both feature selection and model training, with the model settings remaining unchanged. Different feature counts were compared only to examine how performance varied with feature set size, and were not used to replace the DD15 feature set (Section 3.4).

### 2.7. Feature Set Comparison and Discriminative Feature Analysis

After DD15 was defined, it was evaluated at two complementary levels. First, the complete DD15 representation was compared with alternative handcrafted, general-purpose, and pretrained feature sets. Second, the relevance of its individual features was examined across complementary model classes.

*Feature set comparison.* To compare DD15 with alternative feature representations, Random Forest under Method 2 (Original+Augmented/Original) was evaluated using DD15, Literature 14, Extended 50, and eGeMAPS (Table 4). Pretrained wav2vec2-base and HuBERT-base embeddings, together with hybrid DD15+embedding representations, were evaluated under Method 1 (Original/Original) and Method 2. Top-performing configurations are reported in Section 3.5, while detailed comparison is in Supplementary Table S3.

For both wav2vec2-base and HuBERT-base, frame-level representations were extracted from the final transformer layer (“last”) and the fourth transformer layer (“inter”) and temporally mean-pooled. The resulting embeddings were standardized and reduced using principal component analysis (PCA) before classification, using 50 components for embedding-only representations and 10 components for hybrid DD15+embedding representations. Standardization and PCA were fitted using the training data within each cross-validation fold and then applied to the corresponding held-out data. Embedding-only representations were evaluated using Random Forest and logistic regression, while hybrid representations were evaluated using Random Forest, under both Method 1 (Original/Original) and Method 2 (Original+Augmented/Original).

*Discriminative Feature Analysis.* To determine whether the relevance of the individual DD15 features was consistent across complementary model classes, two additional analyses were conducted:

- **SVM feature accumulation.** DD15 features, ordered according to Random Forest SHAP importance, were added incrementally to an RBF-kernel SVM. This analysis assessed how classification performance changed as additional features were introduced and whether a smaller subset retained substantial discriminative information.
- **Logistic regression coefficients.** Absolute logistic regression coefficients provided a linear feature importance measure for comparison with the nonlinear Random Forest Gini and SHAP rankings. Directional agreement between logistic regression and SHAP was quantified as the proportion of features whose values contributed toward the same class. Agreement between feature importance rankings was assessed using Spearman’s ρ.

Both analyses were restricted to DD15 so that the SVM and logistic regression results could be compared directly with the Random Forest Gini and SHAP results used during data-driven selection. Consistency across these complementary analyses was interpreted as evidence that a feature’s discriminative relevance was not specific to a single modelling approach (Section 3.6).

### 2.8. Sensitivity and Confound Analyses

Since all patients were recorded at a single site, while controls were recorded across seven different locations, a possible confound related to the acoustic signature of the recording environment was analyzed using a four-step framework:

- **Site clustering.** K-means clustering (K=7, the number of control recording sites), an unsupervised method that groups recordings according to their acoustic similarity without using site labels, was applied to the control recordings. If the resulting clusters closely matched the actual recording sites, this would suggest that recording environment systematically shaped the acoustic feature space. The silhouette score measured how clearly separated the resulting clusters were (higher values indicate more distinct clusters), while the Adjusted Rand Index (ARI) measured how closely the clusters corresponded to the actual recording sites (higher values indicate stronger agreement, with 1 representing perfect agreement).
- **Per-feature diagnostic vs. site effect.** For each DD15 feature, Cohen’s *d* quantified the standardized difference between patients and controls, computed as *patients − controls*. Larger absolute values indicate greater group separation, while negative values indicate lower values in patients. A Kruskal–Wallis (KW) test was used to assess whether individual feature values differed across the seven control sites. The magnitude of this site effect was summarized using $\eta^{2}$ (eta-squared), an effect-size estimate derived from the KW statistic, with larger values indicating stronger site-related differences.
  As an additional descriptive comparison, we calculated the mean value of each feature across all 41 patients and separately the mean value at each of the seven control sites. The smallest and largest of the seven control site means defined the control site mean range. A feature met the site range separation criterion when the patient group mean fell outside this range, meaning that it was either lower or higher than the average observed at every individual control site. This criterion is distinct from $\eta^{2}$ and does not by itself exclude recording site confounding.
- **Model reliance.** Using mean absolute SHAP values, we calculated how much of the model’s total feature importance came from features meeting the site range separation criterion. This showed whether the classifier relied mainly on features for which the patient group mean lay beyond the range of the control site means.
- **Sensitivity analysis.** Features that combined a relatively large site effect ($\eta^{2}\geq 0.25$) with a patient group mean within the control site mean range were removed, and classification was re-evaluated using the remaining features.

Because the control site groups were small and unequal in size, and because no controls were recorded at the patient site, these analyses were treated as sensitivity checks rather than as a means of eliminating recording site confounding effects.

### 2.9. Additional Analyses

*Clinical correlations.* Spearman rank correlations were computed between Random Forest prediction probabilities and clinical scores: PANSS total, positive, and negative subscales for patients, and CAPS total and PDI total for controls (Section 3.7). These analyses tested whether higher patient class prediction probabilities were associated with symptom severity or anomalous perception scores. In addition, clinical scores were compared between correctly classified and misclassified participants using Mann–Whitney U tests, and permutation testing (10,000 iterations) was used to test whether the clinical-score differences observed between correctly and incorrectly classified participants exceeded what would be expected from random participant subsets of the same size.

*Medication confound.* Chlorpromazine-equivalent (CPZ) dose was compared between correctly and incorrectly classified patients using a Mann–Whitney U test, to test whether medication level was associated with classification outcome (Section 3.7). Partial correlations between prediction probability and PANSS subscales, controlling for CPZ, tested whether any links between model output and symptom severity were affected by the antipsychotic dose.

*AVH subgroup analysis.* Within the patient group (n=41), a secondary classification distinguished between patients with and without history of auditory verbal hallucinations (AVH+ and AVH− patients) (Section 3.7). This subgroup comparison was based on the findings of the parent study, which found a link between auditory verbal hallucination history and differences in self-voice perception [21]. The present analysis therefore tested whether the acoustic features also captured information related to AVH history, beyond the broader patient–control differences.

*Sex stratification.* Model performance was evaluated separately for male (n=45) and female (n=39) subgroups, both to test for sex-specific differences and as a confound check, since several DD15 features are pitch-related and could in principle convey information about the participant’s sex (Section 3.7).

*Exploratory amplitude skewness analysis.* Because amplitude skewness emerged as the highest-ranked discriminative feature, an exploratory source–filter analysis examined whether the patient–control difference was more strongly associated with the estimated glottal source or the vocal-tract filter. The analysis combined LPC (linear predictive coding) inverse filtering, cepstral liftering, direct formant analysis, and spectral tilt analysis; full methodological details are provided in Supplementary Section S1.

*Preprocessing transfer analysis.* As an exploratory sensitivity analysis, the final Random Forest model trained on the standardized 500 ms recordings was applied without retraining to features extracted from the original unprocessed full-length recordings. As an additional recording quality characterization, the RMS level difference between the vocalization and the preceding recorded background segment was calculated after removal of DC offset only, using the original Audacity project files available for 39 of the 41 patients. Recording condition measures were also examined, and the association between amplitude skewness and diagnosis was assessed before and after accounting for RMS and low-frequency energy. Because both preprocessing and segment duration differed between the two input conditions, this analysis was treated as a transfer check rather than as a matched preprocessing ablation. Full methodological details of the transfer analysis are provided in Supplementary Section S4. Additional sensitivity analyses of the processed 500 ms inputs, matched raw-versus-processed segments, and augmentation induced feature changes are reported in Supplementary Section S5.

## 3. Results

### 3.1. Baseline Model Performance

The baseline model performance was established using the Literature 14 and Extended 50 feature sets under Method 1 (Original/Original), in which both the training and test sets contained only original recordings. Performance was evaluated using Random Forest, XGBoost, and logistic regression (Table 5).

**Figure 3:**
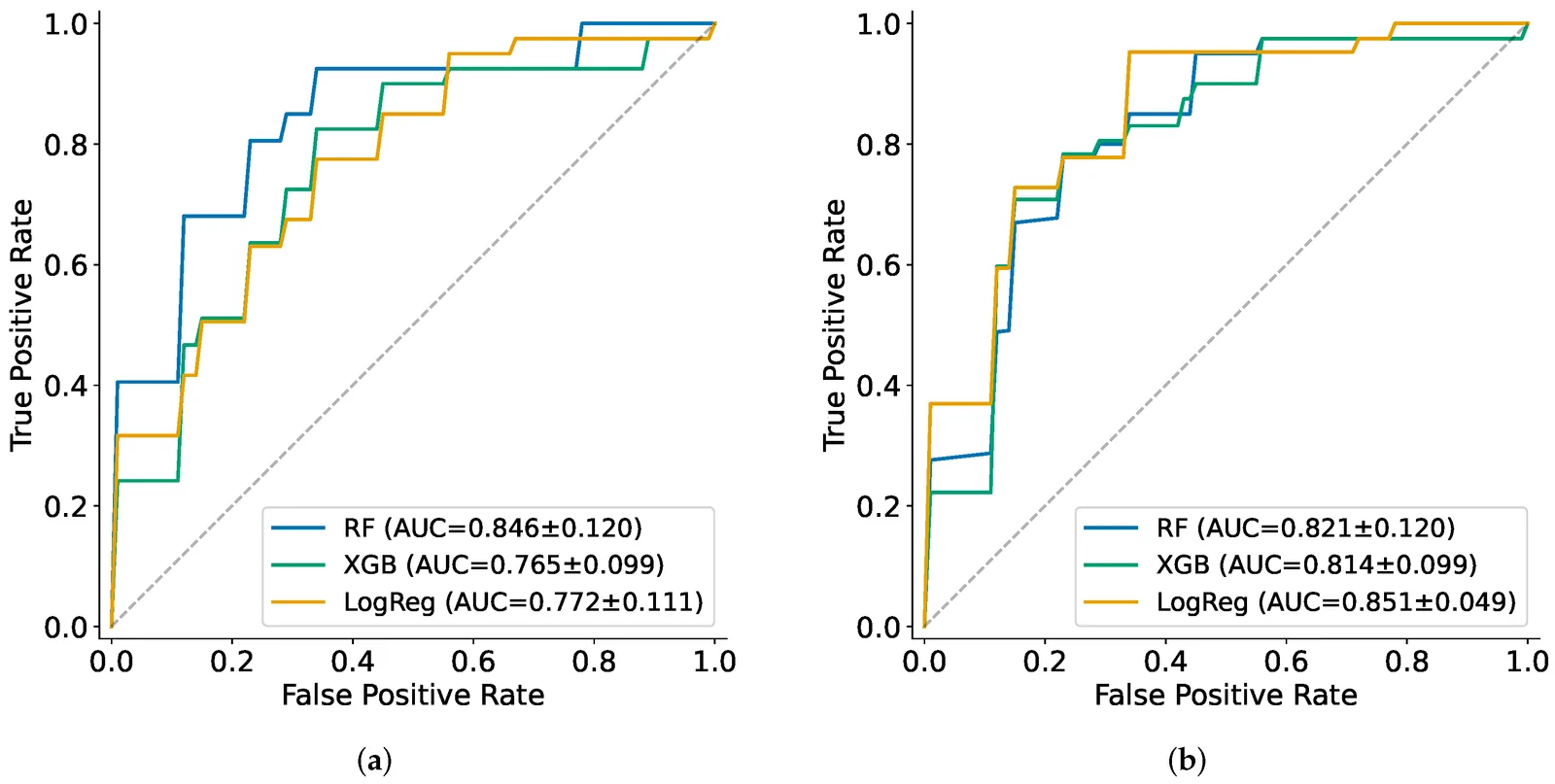
Baseline classification performance, shown as ROC curves for (**a**) Literature 14 and (**b**) Extended 50 feature sets under Method 1 (Original/Original). AUC values in the legends are mean ± SD across five cross-validation folds.

Baseline performance differed between the two feature sets. On Literature 14, Random Forest achieved the highest AUC, whereas on Extended 50, logistic regression performed best. Increasing the feature set from 14 to 50 features improved the performance of logistic regression and XGBoost but did not improve Random Forest. Random Forest consistently achieved the highest sensitivity, while logistic regression produced the highest specificity on the Extended 50 feature set. The corresponding ROC curves are shown in Figure 3.

The ROC curves (Figure 3) illustrate the trade-off between sensitivity and specificity across all classification thresholds. Consistent with the AUC values in Table 5, Random Forest showed the best discrimination on the Literature 14 feature set, whereas logistic regression achieved the highest overall performance on the Extended 50 feature set.

Given the limited sample size and the differences in performance across feature sets and classifiers, we next investigated whether conservative data augmentation could improve classification performance and the robustness of the resulting models.

### 3.2. Effect of Data Augmentation

The effect of data augmentation on model performance was tested with the Extended 50 feature set on Random Forest, XGBoost, and logistic regression (Table 6). Augmentation was evaluated on Extended 50 as the largest, pre-selection feature space with the aim of establishing whether augmentation helps performance at all before any feature selection.

The effect of augmentation varied across classifiers. For Random Forest, mean AUC was numerically higher under Method 2 than Method 1 (0.842 ± 0.102 vs. 0.821 ± 0.120), whereas XGBoost and logistic regression did not show the same pattern. Because these differences were small relative to fold-to-fold variability, they are treated descriptively rather than as evidence of a definitive augmentation gain. Method 3 did not systematically increase performance across models. Random Forest under Method 2 was carried forward because it combined original and augmented training data while retaining evaluation exclusively on original held-out recordings.

### 3.3. Data-Driven Feature Selection

*Gini importance.* Feature selection was performed using Random Forest Gini importance on Extended 50 feature set under Method 2 (Original+Augmented/Original). Random Forest was chosen for interpretability, Method 2 for approximating the real-world deployment, and Extended 50 as the largest candidate pool. Mean Gini importance across folds produced a ranked list of the top 15 features across seven acoustic domains (Table 7).

Amplitude skewness was the most important feature, followed by HNR and local shimmer. The five highest-ranked features accounted for approximately 26% of the total Gini importance, while representing 47% of the overall importance among the selected features. Ten features were selected consistently across at least four folds, while the remaining selected features showed lower selection stability. Although individual feature rankings varied, the overall ranking remained highly consistent (mean Spearman ρ = 0.822), while the top-15 feature subsets showed moderate overlap (mean Jaccard similarity = 0.558).

These fifteen features, spanning distributional, voice quality, cepstral, spectral, chroma, formant, and prosodic domains, were defined as the Data-driven feature set (DD15) and used as the primary feature set for the final model evaluation.

*SHAP analysis.* SHAP was then applied to the same Random Forest model as a complementary interpretation method for the Gini-based feature ranking. It identified the same fifteen DD15 features and provided additional information on the direction of their contributions toward the patient class prediction (Table 8).

**Figure 4:**
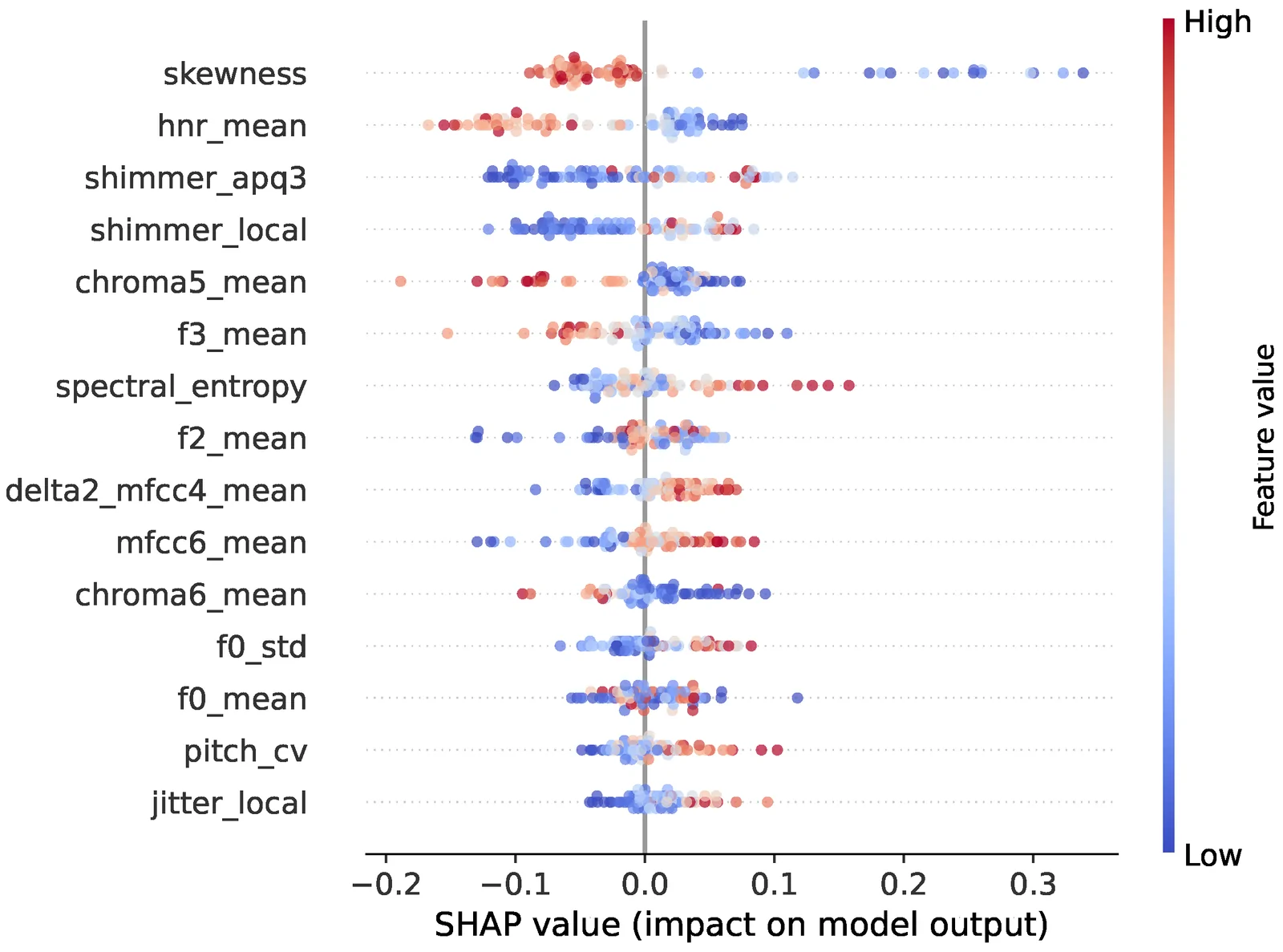
SHAP feature impact on model output (beeswarm) for the DD15 Random Forest model. Colour encodes feature value (blue low, red high); horizontal position is the SHAP value (impact on patient class prediction).

SHAP and Gini rankings showed strong agreement, with only minor differences in feature ordering. This analysis additionally provided directional information, indicating which feature values contributed toward patient class predictions (Figure 4).

Because SHAP identified the same feature set as the Gini ranking on the same Random Forest configuration, it was used as a complementary importance assessment rather than to select additional features. The complete overlap indicates consistency between Gini- and SHAP-based importance assessments within this model.

### 3.4. Final Model Performance

To select the final model using the defined data-driven feature set, all three classifiers and all four methods were evaluated with DD15 (Table 9).

Random Forest achieved the highest AUC under all four training methods, consistently outperforming XGBoost and logistic regression. Performance under Method 4 (Augmented/Original) was comparable to that of Method 2 (Original+Augmented/Original) for Random Forest. Method 2 was retained as the primary configuration because it incorporated both original and augmented recordings during training while evaluating performance exclusively on original recordings, the target data type for this study. Compared with the best-performing baseline model using the Extended 50 feature set (Table 5 and Section 3.1), the fixed DD15 configuration produced a higher point-estimate AUC while reducing the number of input features from 50 to 15. Since DD15 was selected using the complete dataset, this comparison should, however, be interpreted descriptively.

**Figure 5:**
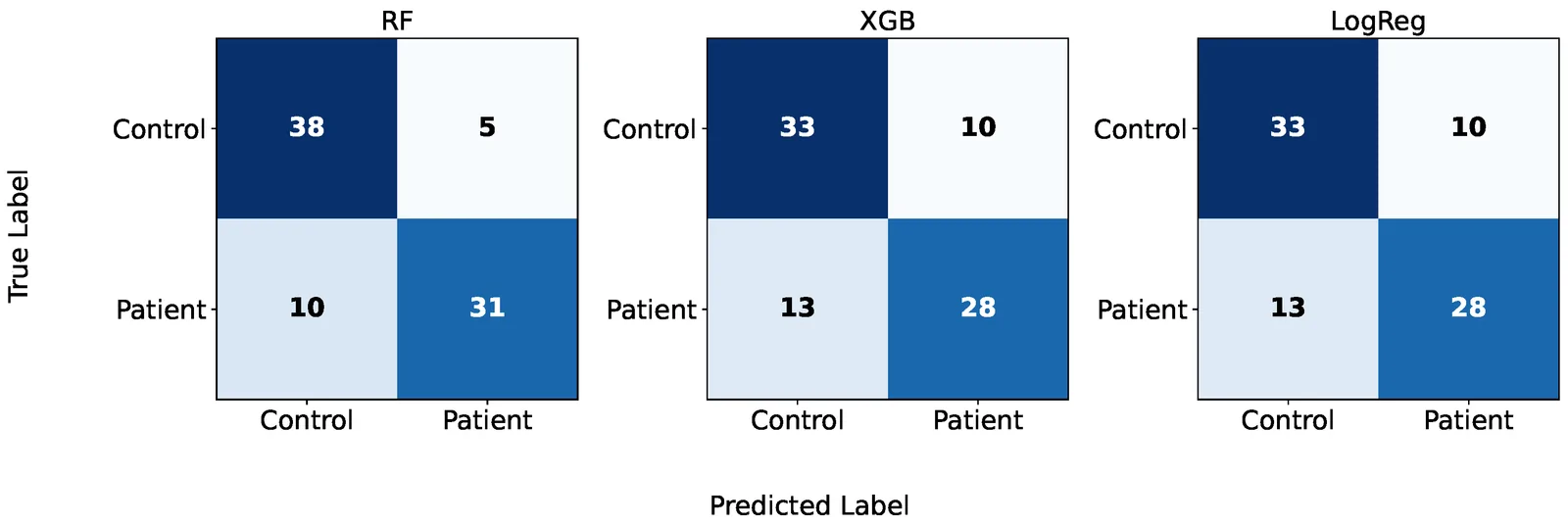
Confusion matrices of all three classifiers with the Data-driven 15 feature set under Method 2 (Original+Augmented/Original), based on pooled participant-level out-of-fold predictions across the five cross-validation folds at the default threshold of 0.50.

The best performing Random Forest on Method 2 generated five false positives among 43 controls (11.6%) and ten false negatives among 41 patients (24.4%), whereas XGBoost and logistic regression resulted in identical confusion matrices, with ten false positives (23.3%) and thirteen false negatives (31.7%) (Figure 5).

**Figure 6:**
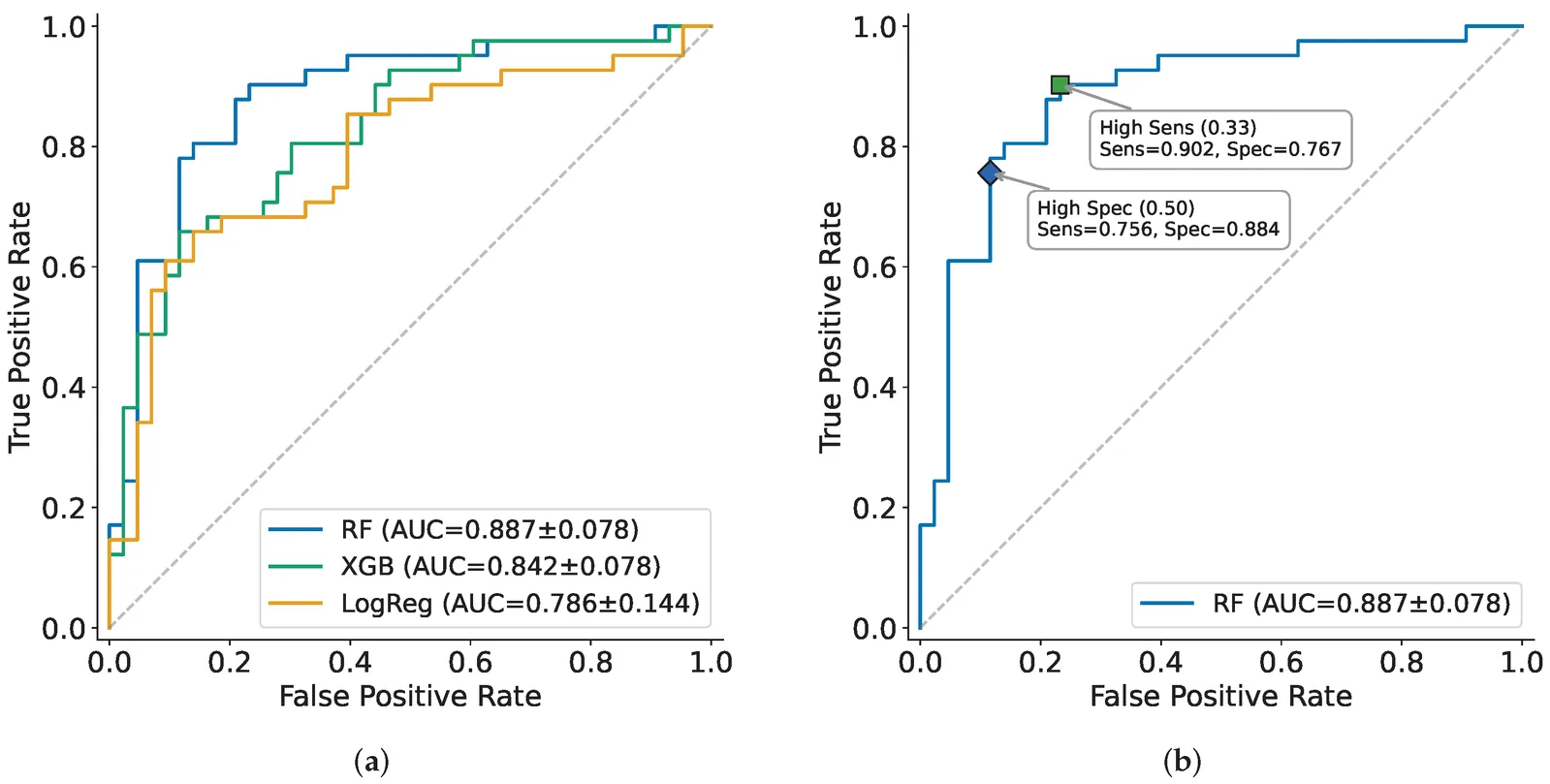
Final model comparison: (**a**) ROC curves for all three classifiers with Data-driven 15 feature set under Method 2 (Original+Augmented/Original); (**b**) Random Forest + DD15 + Method 2 (Original+Augmented/Original) operating points. The ROC curve is based on pooled out-of-fold predictions. AUC values in the legend are mean ± SD across five cross-validation folds.

Using the pooled Method 2 (Original+Augmented/Original) predictions for the Random Forest model, an adjusted screening threshold of t = 0.33 produced a sensitivity of 0.902 and a specificity of 0.767. In comparison, the default threshold of t = 0.50 achieved a sensitivity of 0.756 and a specificity of 0.884 (Figure 6).

Although XGBoost and logistic regression produced identical hard-label classifications at the default threshold of 0.5, their ROC curves and AUC values differed because AUC evaluates the ranking of predicted probabilities across all possible decision thresholds rather than the binary predictions at a single threshold. The operating point shown at t = 0.33 illustrates one possible trade-off between sensitivity and specificity for the final Random Forest model. Because this threshold was selected subsequently using the same pooled out-of-fold predictions on which performance was evaluated, it is shown only as an illustrative sensitivity–specificity trade-off and not as an independently validated operating point.

Because the DD15 feature set was derived from Random Forest Gini importance on the full dataset (Section 2.6), the performance estimates reported above may be optimistic due to this circularity. To address this issue and provide a more realistic performance estimate, an additional five-fold cross-validation with fold-wise feature selection was performed. Feature ranking and selection were repeated independently within each training fold, with the results evaluated using participants held out for testing (Table 10).

The fold-wise feature-selection estimate produced a lower AUC than the fixed DD15 evaluation, as expected once circularity is reduced by excluding held-out participants from feature ranking. Mean AUC peaked at 12 features, but since each fold selected a different subset, this only indicates the approximate dimensionality of the discriminative signal rather than a validated 12-feature set. DD15 is therefore retained as the fixed, final reportable feature set.

### 3.5. Feature Set Comparison

To evaluate whether the data-driven feature selection provided an advantage over alternative feature representations, Random Forest under Method 2 (Original+Augmented/Original) was compared across the DD15, Literature 14, Extended 50, and eGeMAPS feature sets (Table 11).

In the fixed DD15 comparison, DD15 produced the highest numerical AUC (0.887 ± 0.078). Since DD15 was selected using the full dataset, the differences in point estimates cannot be used to demonstrate superiority over representations that were not selected in the same way.

Table 12 compares the final DD15 model with pretrained speech embeddings and hybrid feature representations.

The fold-wise 15-feature estimate (0.837 ± 0.086) was not higher than eGeMAPS (0.861 ± 0.093) and was comparable with HuBERT inter + RF (0.833 ± 0.137). We therefore interpret DD15 primarily as a compact and interpretable representation whose conservative internal performance lies in the same general range as the stronger alternative representations.

### 3.6. Identification of Discriminative Acoustic Features

We next examined whether individual DD15 features showed consistent discriminative value across complementary model classes. Three complementary analyses were conducted: SVM feature accumulation, comparison of SHAP and logistic-regression rankings, and evaluation of directional agreement between models.

SVM feature accumulation analysis provided an additional discriminative assessment of each DD15 feature. Classification with the two highest-ranked DD15 features (amplitude skewness and harmonic-to-noise ratio) already provided substantial discriminative information (AUC = 0.762). Performance improved with the addition of further features, reaching its highest value with 10 features (AUC = 0.853) before declining for the complete DD15 set (AUC = 0.785), consistent with the fold-wise feature selection analysis (Section 2.6 and Table 10) in which mean AUC was numerically highest with 12 selected features (descriptive comparison).

Overall direction agreement between SHAP and logistic regression was 14 of 15 features (93%) (Table 13).

The only mismatch was for spectral entropy, where higher values shifted predictions toward the patient class according to SHAP, whereas for logistic regression higher values shifted predictions towards the control group. The Spearman rank correlation between logistic regression |β| and SHAP |mean| ranks was ρ = 0.143 (*p* = 0.61), indicating low and non-significant association.

Taken together, these analyses indicate that amplitude skewness and HNR were the features most consistently influential across model classes. Shimmer’s strong ranking under Gini and SHAP was not reflected in logistic regression, likely due to the greater sensitivity of tree-based models to nonlinear or threshold effects.

### 3.7. Sensitivity and Confound Assessment

K-means clustering (K=7) applied to the control acoustic features produced a silhouette score of −0.19 and an Adjusted Rand Index of −0.02, indicating that clearly separated clusters corresponding to the seven control recording sites were not recovered in this sample. Given the small and unequal site groups, this result should not be interpreted as evidence that recording site effects were absent. Per-feature recording site effects are summarized in Table 14.

Recording site effects were generally small and should be interpreted descriptively because the control site groups were small and unequal in size. Of the DD15 feature set, mean F0 and MFCC6 showed nominally significant site effects, whereas the two highest-ranked discriminative features—amplitude skewness and HNR—showed relatively small, non-significant site effects.

For seven of the fifteen DD15 features, the mean value across patients lay outside the minimum to maximum range of the seven site-specific control means. In other words, for these features, the patient group mean was more extreme than the mean observed at any individual control site. These features included the three highest-ranked Gini features and collectively accounted for more than half of the total DD15 SHAP importance.

To further assess the influence of recording site effects, three DD15 features showing both relatively large site effects ($\eta^{2}$ ≥ 0.25) and patient means within the control site range (APQ3 shimmer, mean F0, MFCC6) were removed. The resulting DD12 feature set achieved an AUC of 0.850, representing only a small decrease from the original DD15 model (0.887, Table 9 and Section 3.4). The decrease was smaller than the fold-to-fold standard deviation of the DD15 estimate, indicating that classification performance was relatively stable after the removal of these three site-sensitive features.

Additional sensitivity analyses of the processed 500 ms inputs showed that the very large raw RMS difference was substantially reduced after preprocessing, while residual differences remained in absolute DC offset and low-frequency energy (Supplementary Section S5.2.1). The processed segment amplitude skewness association was attenuated after adjustment for these measures and was sensitive to recording-condition variable specification. In a leakage-safe Method 1 sensitivity analysis, within-fold adjustment of DD15 for RMS, absolute DC offset, and low-frequency energy reduced Random Forest AUC from 0.873 ± 0.109 to 0.806 ± 0.089, indicating that the measured recording condition variables contributed to, but did not fully account for the observed internal discrimination. In 64 high-confidence matched raw/processed pairs, several patient-control acoustic differences were already present in the raw source segments (Supplementary Section S5.2.2).

Clinical analyses were used to examine whether classifier confidence or errors were associated with current symptom severity or medication use. PANSS measures schizophrenia symptom severity, while CAPS and PDI characterize anomalous perceptual experiences and delusion-like beliefs. These scales were not treated as alternative diagnostic classifiers, but instead tested whether classifier probabilities and errors were associated with the clinical measures. The chlorpromazine-equivalent (CPZ) dose provides a standardized measure of exposure to antipsychotic medication across different drugs. These analyses test statistical associations and do not establish causal medication effects or clinical diagnostic utility (Table 15).

Prediction probabilities were not significantly associated with PANSS, CAPS, or PDI scores, and CPZ dose did not differ between correctly and incorrectly classified patients. Adjustment for CPZ changed the PANSS correlations only minimally (Δρ ≤ 0.015), providing no indication in this sample that antipsychotic dose materially altered these associations.

Misclassification analyses likewise showed no significant differences in PANSS or CAPS scores between correctly and incorrectly classified participants. The only exception was that the five controls misclassified as patients had lower PDI scores than correctly classified controls. However, given the small subgroup and the number of uncorrected comparisons, this isolated finding is reported descriptively.

*AVH subgroup.* Classification between AVH+ and AVH− patients achieved an AUC of 0.769 ± 0.147, exceeding chance according to permutation testing (*p* = 0.013). However, classification accuracy remained close to the majority-class baseline, indicating limited practical separability between AVH subgroups.

*Sex-stratified analysis.* Performance was higher in females than males across all evaluation metrics (Table 16). Because subgroup models were trained and cross-validated independently within each sex and the female subgroup was relatively small, particularly with perfect specificity among controls, these findings should be interpreted cautiously.

*Exploratory amplitude skewness analysis.* Amplitude skewness showed only weak correlations with HNR, shimmer, and jitter, indicating that it captures information not already represented by these established voice quality measures. Exploratory source–filter analyses consistently associated the amplitude skewness difference more strongly with the vocal-tract filter than with the estimated glottal source. Full results, including the formant bandwidth findings, are reported in Supplementary Section S1.

*Preprocessing transfer analysis.* As an additional characterization of recording quality, the vocalization-to-background RMS level difference could be calculated for 39 patients for whom the original Audacity project files were available. The median difference was 34.63 dB (IQR 32.10–37.82 dB; mean 34.40 ± 5.47 dB; range 20.47–45.68 dB). This represents a relative level difference within the digital recordings rather than a calibrated acoustic SNR.

Direct application of the final model to the original unprocessed full-length recordings resulted in substantially lower performance (AUC = 0.639, 95% CI 0.514–0.763; Supplementary Section S4). Amplitude skewness in these recordings was associated with diagnosis (r = −0.345, *p* = 0.002), but the association decreased after accounting for RMS and low-frequency energy (r = −0.042, *p* = 0.71), indicating sensitivity of the raw measurement to recording conditions. Because both preprocessing and segment duration differed from the main analysis, this analysis does not isolate the effect of preprocessing itself.

## 4. Discussion

The aim of this study was to examine whether short vowel vocalizations carry sufficient acoustic information to separate schizophrenia patients from healthy controls. We developed a machine learning classification pipeline based on domain-specific acoustic features and compared different classifiers, training strategies, and feature representations. A compact subset of features emerged as most important across different methods, indicating that classification relied on a limited number of voice quality and spectral characteristics. Sensitivity analyses did not indicate that performance was primarily explained by medication or symptom-related effects, while recording-site confounding cannot be excluded with the present dataset.

### 4.1. Model Performance and Data Augmentation

The first goal was to establish a baseline performance. Three classifiers (Random Forest, XGBoost, logistic regression) were trained on two feature sets extracted from 500 ms voice recordings: a literature-derived 14-feature set and an extended 50-feature set. Even without augmentation or feature selection, both baselines achieved meaningful discrimination, indicating that sustained phonation carries group-discriminative acoustic information (Table 5 and Figure 3).

Augmentation produced small, model-dependent numerical changes rather than a uniform performance gain (Table 6). For Random Forest, Method 2 (Original+Augmented/Original) produced a higher mean AUC than Method 1 (Original/Original) on Extended 50 (0.842 vs. 0.821) and on fixed DD15 (0.887 vs. 0.873), but these differences were small relative to fold-level variability. Feature-level analysis further showed that augmentation materially altered several micro-acoustic measures, particularly shimmer and HNR (Supplementary Section S5.2.3). Augmented copies should therefore be viewed as synthetic training perturbations rather than as acoustically equivalent repeated recordings. Under Method 2, these perturbations were used only for training, while evaluation remained on original held-out recordings (Table 9). Importantly, Method 3 (Augmented/Augmented) did not produce systematically higher performance than Method 1, suggesting that augmentation did not generally inflate performance in these comparisons. One possible explanation for the model-specific pattern is that the augmented recordings may have acted as implicit regularization for Random Forest and, to a lesser extent, for XGBoost under Method 4 (Augmented/Original). In contrast, the highly correlated augmentation variants provided little additional information to the already regularized logistic regression and may instead have contributed mainly as added noise, thus reducing performance. Since each participant contributed only one original 500 ms segment, augmentation should not be interpreted as reproducing genuine within-participant voice variability.

Method 2 was retained as the primary augmentation configuration for subsequent Random Forest analyses because it incorporated synthetic training variation while evaluating performance only on original held-out recordings. Random Forest with DD15 under Method 2 achieved the highest point-estimate AUC (0.887), accuracy, specificity, and F1 score among the evaluated model-method combinations (Table 9). When feature selection was repeated independently within each training fold, the corresponding 15-feature estimate was 0.837, while the mean AUC was highest at 0.858 with 12 features selected (Table 10). Accordingly, the fixed DD15 AUC of 0.887 should be regarded as an exploratory estimate, whereas the 0.837 estimate from fold-wise feature selection provides a more conservative indication of performance when feature ranking is separated from the held-out participants. Both of these estimates remain internal to the current study groups, and model selection, augmentation choice, feature set comparisons, and threshold exploration were not evaluated in an independent dataset. These more conservative estimates account for a circularity in the original evaluation. Since DD15 was selected using Random Forest Gini importance on the full dataset, testing Random Forest on DD15 directly could overstate the performance. When features were instead reselected independently within each training fold via fold-wise evaluation, the model still demonstrated substantial discrimination on participants who were excluded from both feature selection and training.

### 4.2. Data-Driven Feature Set Performance

Having defined DD15 as the final feature set and addressed the selection-related performance boost, we next compared this task-specific 15-feature representation with larger and more generic alternatives.

Derived from Random Forest Gini importance and supported by the complementary SHAP analysis (Table 7 and Table 8 and Figure 4), DD15 produced higher point-estimate performance than the Literature 14 and Extended 50 representations under the same Random Forest + Method 2 configuration, despite using less than a third of the Extended 50 features (Table 11). However, because DD15 was selected using the complete dataset, this fixed comparison is optimistic and should not be interpreted as establishing superiority. Rather, it shows that a compact, task-specific subset can retain strong internal discrimination while remaining substantially more interpretable than the full feature pool.

Against representations not derived from this dataset, fixed DD15 also achieved the highest point-estimate AUC among the evaluated feature sets, including eGeMAPS and the tested wav2vec2 and HuBERT configurations (Table 12). However, this comparison is likewise affected by the selection dependence of the fixed DD15 estimate. When feature selection was repeated within the training folds, the 15-feature estimate was 0.837 ± 0.086, which was not higher than eGeMAPS (0.861 ± 0.093) and was comparable with HuBERT inter + RF (0.833 ± 0.137). The present data therefore do not establish that DD15 outperforms these alternative representations.

Taken together, these results indicate that DD15 provides a compact and interpretable acoustic representation with internal performance in the same general range as the stronger alternative representations evaluated here. Its small dimensionality and direct acoustic interpretability remain useful in the current feasibility context and make it a tractable candidate representation for prospective validation.

### 4.3. Discriminative Acoustic Features

To determine which DD15 features contributed most consistently to classification, we compared feature relevance across complementary model classes rather than relying on a single ranking method.

DD15 showed moderate-to-high selection stability: 7/15 features appeared in all five folds and 10/15 in at least four folds (Table 7). When feature selection was repeated independently within each training fold, mean AUC was numerically highest with 12 selected features (Table 10), suggesting that much of the discriminative information could be retained with slightly fewer than 15 features. However, this comparison was descriptive and did not define a single validated 12-feature set.

A similar pattern emerged from the complementary model analyses. The SVM feature accumulation curve peaked with ten SHAP-ranked features, supporting the interpretation that most of the useful discriminative information was concentrated within a compact subset of approximately 10–15 features. Logistic regression agreed with SHAP on classification direction for 14 of the 15 features but differed in rank ordering (Table 13), suggesting that the models captured similar directional patterns while assigning different importance to individual features.

Amplitude skewness was the most consistently dominant feature across the Gini, SHAP and logistic regression analyses, whereas HNR and shimmer were the leading voice quality contributors in the final Random Forest model. HNR reflects the balance between periodic harmonic and aperiodic components of the voice signal, while shimmer quantifies cycle-to-cycle variation in amplitude, and both are commonly used as indicators of phonatory stability and voice quality [33]. In the present analysis, lower HNR and higher shimmer values shifted predictions toward the patient class, a pattern consistent with reduced periodicity and greater amplitude instability of the phonatory signal. This direction is also consistent with the sustained-vowel findings of Mouratai et al. [14], who reported lower HNR and higher shimmer in patients with schizophrenia than in healthy controls. However, findings across schizophrenia studies have not been consistent [12,13,14], and these acoustic patterns should not be interpreted as evidence of a specific underlying physiological abnormality.

Amplitude skewness is notable because it is not a standard focus in schizophrenia voice research. Unlike jitter and shimmer, which describe cycle-to-cycle perturbation, skewness summarizes the asymmetry of the amplitude distribution across the entire waveform segment. Its weak correlations with HNR, shimmer, and jitter therefore suggest that it captures information that is not already represented by these voice quality properties. In the exploratory source–filter decomposition, the group difference was more consistent with a vocal-tract filter contribution than with a glottal-source change. This mechanistic interpretation is still preliminary, and requires validation using standardized recording and preprocessing across independent sites. Further details, including the formant-bandwidth findings, are provided in Supplementary Section S1.

The matched raw-versus-processed analysis provides additional context for these findings at the feature level. Jitter, HNR, and amplitude skewness demonstrated a strong degree of correspondence between the raw and processed data, whereas shimmer measures showed a more moderate correspondence. HNR also showed a larger processing induced decrease in patients than in controls. Importantly, patient-control differences in amplitude skewness, jitter, local shimmer, and HNR were already present in the matched raw source segments, indicating that preprocessing did not create these differences anew, although preprocessing altered their magnitudes, particularly for shimmer measures and HNR. These results support treating the leading acoustic measures as candidate features rather than as processing-invariant or mechanistically specific markers (Supplementary Section S5.2.2).

Overall, a stable core of seven features (amplitude skewness, HNR, shimmer local, shimmer APQ3, Chroma 5, spectral entropy, F0 mean) appeared in all five fold-specific top 15 lists. Together with the cross-model prevalence of amplitude skewness, this stability supports the importance of standardized feature extraction and reporting, especially considering the recognized inconsistencies between acoustic toolkits [32].

### 4.4. Confounds, Clinical Correlates, and Subgroups

These confound analyses evaluated whether the performance of the model could be influenced by the recording environment, medication or symptom state, rather than by vocal differences linked to diagnosis.

The recording-site analyses should be viewed as sensitivity analyses, not as proof that site-related confounding was absent. The clustering analysis did not identify clearly distinct clusters aligned with the seven control recording locations, and the feature-wise analyses showed mostly modest differences across those control sites. For seven DD15 features, including the two top-ranked features, amplitude skewness and HNR, the mean patient values also fell outside the range of means observed across the seven control sites. Moreover, excluding the three DD15 features with the strongest combination of control site variability and overlap with the patient mean (APQ3 shimmer, mean F0, MFCC6) led to only a small drop in classification performance. Together, these results suggest that the measured variability across the available control sites does not straightforwardly explain the overall pattern of patient–control differences. However, the control site groups were small and imbalanced, and no healthy controls were recorded in the patient testing room. As a result, diagnosis and recording environment are confounded in this dataset, and an influence of site-specific room acoustics cannot be ruled out. This limitation is particularly relevant for features such as HNR and shimmer, which are known to be sensitive to recording conditions [41,42,43].

Analyses of the actual processed 500 ms inputs further showed that preprocessing largely removed the very large raw RMS difference but left residual differences in absolute DC offset and low-frequency energy. Leakage-safe within-fold adjustment of DD15 for these three measures reduced Method 1 AUC from 0.873 to 0.806. Thus, adjustment for these measured recording condition variables reduced, but did not eliminate, the observed internal discrimination. Because all patients were recorded in one room, these sensitivity analyses cannot rule out the possibility of unmeasured or nonlinear patient-site effects (see Supplementary Section S5.2.1).

Clinical and medication analyses showed no systematic association between prediction probability and current symptom severity, and CPZ dose did not differ between correctly and incorrectly classified patients. AVH subgroup classification was above chance but showed limited practical separability. Together, these results suggest the model was not primarily tracking symptom severity in this sample—unlike studies using reading or semi-structured speech, where acoustic features have been linked to negative symptom severity or used to distinguish positive and negative symptom profiles [12,15]. This difference may reflect the sustained vowel task’s narrower phonatory focus, which strips out most linguistic and connected speech information. One exception was lower PDI scores among false-positive controls, reported descriptively given the small subgroup and multiple comparisons (Table 15).

Finally, since DD15 includes pitch-related features, sex-stratified evaluation served as an additional confound check. Performance remained high in both subgroups (Table 16), though these estimates should be interpreted cautiously given the limited sample size.

### 4.5. Limitations

This study has several limitations. First, the sample size (41 patients, 43 controls) is small for machine-learning evaluation, resulting in wide fold-to-fold variability and limited precision of performance estimates. As a consequence, small numerical differences between classifiers, feature sets, augmentation conditions, and feature ablation analyses should be interpreted cautiously rather than as definitive performance rankings.

Second, the recording design was imbalanced by site, with all patients recorded at a single clinical site and controls recorded across seven other sites. The control site groups were small and unequal, limiting the strength of comparisons among recording locations. Since no controls were recorded in the patient testing room and no patients were recorded across the control sites, it is not possible to separate diagnosis from the patient recording environment in the present dataset. Accordingly, the site analyses can only provide sensitivity checks against the variation measured among the control locations, and cannot rule out a contributing factor from the patients’ recording environment. A balanced multi-site design is therefore required to more fully address environmental confounding and test generalization [13,44].

Finally, all recordings underwent the same peak normalization, noise reduction, and manual selection of a stable 500 ms segment for patients and controls, as part of the parent study [21]. These steps were used to provide more standardized stimuli for the voice perception task and to reduce differences in loudness and background noise across participants and recording locations. Nevertheless, preprocessing may alter acoustic detail, including perturbation measures such as jitter and shimmer, while selection of the most stable portion restricts the analysis to a deliberately low variability segment. This limits the direct interpretation and transferability of individual acoustic features. The exploratory analysis of the original unprocessed full-length recordings further showed limited transfer of the classifier and sensitivity of raw amplitude skewness to recording conditions (Supplementary Section S4). Because both preprocessing and segment duration differed in that analysis, the specific contribution of preprocessing cannot be isolated. A matched raw-versus-processed sensitivity analysis was additionally available for 64 high-confidence pairs and confirmed that preprocessing can alter the magnitude of individual acoustic measures.

Additionally, model hyperparameters were fixed rather than tuned, and unmeasured confounding factors such as education level, illness duration, and recording quality cannot be ruled out. The patient group was also not designed to represent different stages of schizophrenia, and individuals experiencing an acute episode were excluded. The present results should therefore not be interpreted as a classification of disease stage nor should they be considered applicable to different stages of illness.

### 4.6. Future Work

Future work should test the proposed approach prospectively in larger, balanced, multi-site and multilingual cohorts in which both diagnostic groups are represented across recording environments, using standardized recording and preprocessing procedures. Larger datasets should also evaluate formal hyperparameter optimization using nested cross-validation or independent validation data. From a biomedical signal perspective, the brief sustained vowel protocol is attractive because it requires minimal acquisition time and can be implemented using widely available commercial microphones. The compact DD15 representation could facilitate both lightweight processing and transparent model assessment. Deployment-oriented studies should therefore explicitly examine device and microphone mismatch, background noise, and performance under minimally processed recording conditions.

Longitudinal and repeated measures designs are needed to accurately assess the stability of the acoustic features within participants. Recording both sustained phonation and connected speech within the same group of participants would provide further insight into the relative contributions of phonatory, prosodic, linguistic and temporal information. Standardized cross-toolkit feature reporting would improve reproducibility [32], while multimodal approaches integrating acoustic, linguistic, and orofacial information could improve clinical specificity [45,46,47,48]. These steps are necessary before sustained vowel analysis can be considered as an accessible component of voice-based screening tools.

### 4.7. Broader Implications and Impact

The central contribution of this study is the finding that a 500 ms sustained vowel carries meaningful group-discriminative acoustic information. Most previous voice-based schizophrenia classifiers have relied on connected speech, semi-structured interviews, sentence reading, or combined acoustic and semantic representations [8,9,20]. In these tasks, phonation is inseparable from linguistic content, pausing, connected-speech prosody, and speaking style. The present results show that meaningful internal discrimination is still possible when these sources of information are substantially reduced, suggesting that group-related acoustic information is also detectable during minimal phonatory tasks.

Although differences in samples, tasks, and evaluation procedures across studies limit direct comparison, the discrimination achieved here was generally comparable to that of recent models using semi-structured interviews, combined acoustic-semantic features, and sentence-reading tasks [15,16,17]. Rather than replacing connected speech analysis, sustained phonation offers a complementary, language-light task that isolates a more specific aspect of vocal production. It also requires minimal acquisition time and can be recorded with widely available microphones, consistent with the established use of sustained vowel tasks in other clinical voice applications [49,50].

A compact and interpretable representation such as DD15 may therefore provide a practical basis for further low-burden voice-sensing research. At this stage, however, the present study is best viewed as a feasibility and hypothesis generating analysis rather than validation of a deployable clinical classifier. Amplitude skewness, HNR, shimmer, and the other DD15 features should be treated as candidate acoustic features for prospective replication under standardized, site balanced recording conditions. With appropriate external validation, such approaches could support more accessible mental health screening [10], though they should be viewed as potential additions to clinical assessment rather than replacements for diagnostic interviews or in-depth speech based evaluations.

More generally, the sustained phonation approach may have applications beyond schizophrenia. Low-level voice alterations have been reported in various psychiatric and neurological conditions, including depression, Parkinson’s disease and amyotrophic lateral sclerosis [51,52,53]. This suggests that the same brief and language-light task could potentially reveal different condition-specific acoustic profiles rather than a single universal voice biomarker. At a broader mechanistic level, voice and self-voice processing involve the interaction of auditory, motor control, multisensory, memory, and self-related processes, several of which have been implicated across different clinical conditions [54]. Therefore, identifying distinct voice acoustic features across different disorders could provide complementary information about which aspects of vocal production and control are most affected by each condition.

## 5. Conclusions

This feasibility study demonstrates that a 500 ms sustained vowel contains meaningful acoustic information to distinguish patients with schizophrenia from healthy controls.

Meaningful discrimination was already present in the baseline models, showing that the signal did not depend on data augmentation or data-driven feature selection. Augmentation produced small, model-dependent numerical changes rather than a uniform performance gain and is therefore best regarded as a synthetic training perturbation.

The fixed DD15 configuration produced the highest point-estimate AUC among the evaluated representations, but this estimate is selection-dependent and does not establish superiority over the alternative representations, while the more conservative fold-wise 15-feature estimate was broadly comparable with the stronger alternatives. Much of the discriminatory information was concentrated in a subgroup of acoustic features, particularly amplitude skewness, harmonic-to-noise ratio, and shimmer. Amplitude skewness was the single strongest discriminator and, unlike jitter and shimmer, is not a standard focus of this literature, since it summarizes the shape of the waveform’s amplitude distribution rather than cycle-to-cycle perturbation. The exploratory source–filter analysis further suggested that the amplitude skewness difference may be associated more strongly with vocal-tract filtering than with the estimated glottal source.

Sensitivity analyses did not indicate that effects related to medication, symptoms, or sex primarily explained classification performance, while the recording design does not allow the patient recording site effect to be separated from diagnosis. Adjustment for RMS, absolute DC offset, and low-frequency energy reduced the model performance but did not eliminate the patient-control discrimination.

Overall, these findings support sustained phonation as a promising language-light and low-burden signal for schizophrenia voice research. Larger, balanced, multi-site studies with standardized recording and preprocessing are now required to determine its generalizability and potential role alongside clinical and connected-speech assessment.

## Figures and Tables

**Table 1:** Participant demographics. Mean values with standard deviation are presented where applicable. Between-group *p*-values are from one-way ANOVA (continuous variables) and Pearson’s chi-square test with Yates’ correction (sex). Chlorpromazine-equivalent dose is reported as CPZ.

	Patients (n=41)	Controls (n=43)	*p*-Value
Age (years)	35.12 ± 9.41	35.53 ± 9.24	0.840
Sex (M/F)	24/17	21/22	0.501
PANSS total	74.20 ± 17.27	—	—
PANSS positive	15.20 ± 4.82	—	—
PANSS negative	23.00 ± 6.28	—	—
CPZ equiv. (mg)	560.41 ± 396.12	—	—
CAPS total	—	4.65 ± 3.54	—
PDI total	—	3.95 ± 2.22	—

**Table 2:** Representative acoustic feature families used in schizophrenia and psychiatric voice research.

Domain	Feature	Description	Repr. Use ^†^
Prosodic	F0 mean	Mean fundamental frequency (perceived pitch).	[11,12,17]
Prosodic	F0 SD/range	Pitch variability/range; reduced variation linked to aprosody and negative symptoms.	[12,32]
Voice quality	Jitter (local)	Cycle-to-cycle frequency perturbation; perceived hoarseness.	[12,17,33]
Voice quality	Shimmer (local)	Cycle-to-cycle amplitude perturbation; perceived roughness.	[12,17,33]
Voice quality	HNR	Harmonic-to-noise ratio; periodic vs. aperiodic energy.	[12,32,33]
Voice quality	CPP/CPPS	Cepstral peak prominence; robust overall dysphonia measure.	[34]
Formant	F1–F5 means	Vocal-tract resonance frequencies; vowel quality and timbre.	[17,32]
Formant	Formant bandwidths	Resonance bandwidths; vocal-tract damping.	[20]
Cepstral	MFCC_1_–*n*/delta-MFCCs	Spectral-envelope shape and its temporal dynamics.	[12,17,32]
Spectral	Spectral centroid/entropy	Spectral centre of mass; spectral flatness/disorder.	[15,17]
Spectral	Spectral tilt/H1–H2	Spectral slope; glottal-source correlate.	[15]
Intensity	Intensity/loudness	Vocal energy; reduced vocal intensity in negative symptoms.	[17]

^†^ Repr. use = representative examples of use in schizophrenia-spectrum speech/voice research.

**Table 3:** Overview of methods with train–test configurations used for data augmentation analyses.

Method	Training Set	Test Set	Purpose
Method 1	Original data	Original data	Baseline classification performance using only original voice recordings.
Method 2	Original data + augmented data	Original data	Evaluates whether adding augmented recordings to the training set improves performance on original recordings.
Method 3	Augmented data	Augmented data	Assesses whether training and testing on augmented recordings artificially inflates classification performance.
Method 4	Augmented data	Original data	Assesses whether a model trained exclusively on augmented recordings generalizes to original recordings.

**Table 4:** Feature representations compared with DD15 under Random Forest, Method 2 (Original+Augmented/Original).

Family	Description
Handcrafted (study-specific)	Literature 14: literature-derived 14-feature set used in prior schizophrenia voice research; Extended 50: full 50-feature candidate pool defined for the present study.
Standardized general-purpose	eGeMAPS: extended Geneva Minimalistic Acoustic Parameter Set, comprising 88 handcrafted temporal, spectral, and energy features and widely used as an interpretable baseline in clinical and affective voice research [19].
Deep learning–based	wav2vec2-base and HuBERT-base: frozen embeddings from self-supervised speech models pretrained on large speech corpora. Hybrid representations combined DD15 with each embedding set [39,40].

**Table 5:** Baseline classification performance on Literature 14 and Extended 50 feature sets under Method 1 (Original/Original). AUC is reported as mean ± SD; all other metrics are mean. Bold indicates the best value per metric.

Feature Set	Model	AUC	Acc.	Sens.	Spec.	F1 Score
	Logistic Regression	0.772±0.111	0.668	0.631	0.698	0.643
Literature 14	Random Forest	0.846±0.120	**0.799**	**0.875**	**0.721**	**0.809**
	XGBoost	0.765±0.099	0.715	0.706	**0.721**	0.707
	Logistic Regression	0.851±0.049	0.762	0.731	**0.794**	0.748
Extended 50	Random Forest	0.821±0.120	**0.810**	**0.853**	0.765	**0.812**
	XGBoost	0.814±0.099	0.762	0.731	0.787	0.751

**Table 6:** Classification performance (AUC) across data augmentation configurations. Values are reported as mean ± SD. Bold indicates the best result per model.

Method	Random Forest	XGBoost	Log. Reg.
Method 1 (Original/Original)	0.821 ± 0.120	0.814 ± 0.099	**0.851 ± 0.049**
Method 2 (Original+Augmented/Original)	**0.842 ± 0.102**	0.806 ± 0.089	0.810 ± 0.081
Method 3 (Augmented/Augmented)	**0.823 ± 0.090**	0.776 ± 0.073	0.790 ± 0.098
Method 4 (Augmented/Original)	**0.826 ± 0.083**	0.826 ± 0.071	0.796 ± 0.122

**Table 7:** Top 15 features selected by Random Forest Gini importance under Method 2 (Original+Augmented/Original). Importance is the mean Random Forest Gini impurity decrease across the five cross-validation folds. Stability describes selection frequency across folds. Values are on the native Gini scale and sum to 1 across all Extended 50 features.

Rank	Feature	Importance	Stability (Folds)	Domain
1	amplitude skewness	0.0766	Core (5/5)	Distributional
2	hnr_mean	0.0475 *	Core (5/5)	Voice quality
3	shimmer_local	0.0475 *	Core (5/5)	Voice quality
4	shimmer_apq3	0.0450	Core (5/5)	Voice quality
5	chroma5_mean	0.0412	Core (5/5)	Chroma
6	spectral_entropy	0.0390	Core (5/5)	Spectral
7	f3_mean	0.0321	Near-core (4/5)	Formant
8	delta2_mfcc4_mean	0.0316	Variable (3/5)	Cepstral
9	chroma6_mean	0.0290	Variable (3/5)	Chroma
10	f0_std	0.0286	Near-core (4/5)	Prosodic
11	f0_mean	0.0281	Core (5/5)	Prosodic
12	f2_mean	0.0276	Variable (3/5)	Formant
13	mfcc6_mean	0.0265	Near-core (4/5)	Cepstral
14	jitter_local	0.0247	Unstable (2/5)	Voice quality
15	pitch_cv	0.0246	Unstable (2/5)	Prosodic

* HNR and local shimmer received near-identical mean Gini importance (unrounded 0.04752 and 0.04749); the 2nd/3rd ordering should be regarded as effectively tied.

**Table 8:** Agreement between Gini importance and SHAP rankings for the DD15 features (including SHAP magnitude and direction).

Feature	Gini Rank	SHAP Rank	Δ Rank	Mean |SHAP|	Direction (SHAP)
amplitude skewness	1	1	=	0.0813	Lower → patient
hnr_mean	2	2	=	0.0620	Lower → patient
shimmer_local	3	4	−1	0.0489	Higher → patient
shimmer_apq3	4	3	+1	0.0597	Higher → patient
chroma5_mean	5	5	=	0.0369	Lower → patient
spectral_entropy	6	7	−1	0.0329	Higher → patient
f3_mean	7	6	+1	0.0349	Lower → patient
delta2_mfcc4_mean	8	9	−1	0.0289	Higher → patient
chroma6_mean	9	11	−2	0.0239	Lower → patient
f0_std	10	12	−2	0.0230	Higher → patient
f0_mean	11	13	−2	0.0218	Lower → patient
f2_mean	12	8	+4	0.0303	Higher → patient
mfcc6_mean	13	10	+3	0.0288	Higher → patient
jitter_local	14	15	−1	0.0202	Higher → patient
pitch_cv	15	14	+1	0.0207	Higher → patient

**Table 9:** DD15 classification results across four training methods and three classifiers. AUC is reported as mean ± SD; all other metrics are mean. Bold indicates the best value per metric.

Method	Model	AUC	Acc.	Sens.	Spec.	F1 Score
Method 1	Random Forest	0.873±0.109	0.786	**0.828**	0.743	0.791
	XGBoost	0.796±0.058	0.773	0.756	0.781	0.765
	Logistic Regression	0.823±0.142	0.737	0.686	0.787	0.718
Method 2	Random Forest	0.887±0.078	**0.823**	0.753	**0.889**	**0.799**
	XGBoost	0.842±0.078	0.725	0.686	0.765	0.704
	Logistic Regression	0.786±0.144	0.725	0.683	0.759	0.708
Method 3	Random Forest	0.851±0.067	0.747	0.713	0.776	0.732
	XGBoost	0.792±0.086	0.684	0.652	0.715	0.662
	Logistic Regression	0.788±0.142	0.711	0.700	0.718	0.706
Method 4	Random Forest	0.886±0.085	0.786	0.706	0.860	0.759
	XGBoost	0.824±0.092	0.737	0.639	0.838	0.697
	Logistic Regression	0.780±0.148	0.738	0.658	0.810	0.709

**Table 10:** Cross-validation performance with fold-wise feature selection by feature count. AUC with fold-wise selection is reported as mean ± SD across folds. Bold indicates the highest mean.

*N* Features	AUC with Fold-Wise Selection
8	0.813 ± 0.112
10	0.829 ± 0.123
12	**0.858 ± 0.096**
15	0.837 ± 0.086

**Table 11:** Feature set comparison under Random Forest, Method 2 (Original+Augmented/Original) (DD15 vs. Literature 14, Extended 50, eGeMAPS). Bold: best values per metric. DD15 was selected using the full dataset, and comparisons involving it should be interpreted accordingly.

Feature Set	*N*	AUC	Acc.	Sens.	Spec.	F1 Score
Data-driven 15	15	**0.887 ± 0.078**	**0.823**	**0.753**	**0.889**	**0.799**
Literature 14	14	0.768 ± 0.101	0.668	0.536	0.800	0.597
Extended 50	50	0.842 ± 0.102	0.775	0.678	0.860	0.737
eGeMAPS	88	0.861 ± 0.093	0.726	0.658	0.787	0.694

**Table 12:** Pretrained embedding results compared with the final model configuration (all under Method 2, Original+Augmented/Original). AUC is reported as mean ± SD. Bold indicates the best value per metric. DD15 was selected using the full dataset, and comparisons involving it should be interpreted accordingly.

Configuration	AUC
DD15+RF	**0.887 ± 0.078**
wav2vec2 last+LR	0.681 ± 0.102
wav2vec2 inter+RF	0.745 ± 0.083
HuBERT inter+RF	0.833 ± 0.137
Hybrid DD15+wav2vec2 RF	0.862 ± 0.103
Hybrid DD15+HuBERT RF	0.862 ± 0.104

**Table 13:** Comparison of DD15 feature importance ranks across Random Forest Gini, SHAP, and logistic regression. The direction comparison is between SHAP and logistic regression. Ranks agree closely between the two tree-based methods and diverge from the linear model (Spearman ρ = 0.143, *p* = 0.61).

Feature	Gini	SHAP	Log. Reg.	Direction
amplitude skewness	1	1	1	Lower → patient
hnr_mean	2	2	5	Lower → patient
shimmer_local	3	4	12	Higher → patient
shimmer_apq3	4	3	15	Higher → patient
chroma5_mean	5	5	7	Lower → patient
spectral_entropy	6	7	13	Mixed *
f3_mean	7	6	2	Lower → patient
delta2_mfcc4_mean	8	9	4	Higher → patient
chroma6_mean	9	11	10	Lower → patient
f0_std	10	12	9	Higher → patient
f0_mean	11	13	14	Lower → patient
f2_mean	12	8	11	Higher → patient
mfcc6_mean	13	10	3	Higher → patient
jitter_local	14	15	8	Higher → patient
pitch_cv	15	14	6	Higher → patient

* Mixed: SHAP higher → patient, logistic regression higher → control.

**Table 14:** Diagnostic effect sizes and recording site effects for DD15 features. Cohen’s *d* was computed as patients minus controls. Site $\eta^{2}$ and the Kruskal–Wallis *p*-value describe variation among the seven control sites. The final column indicates whether the mean feature value across all patients lay outside the minimum-to-maximum range of the seven site-specific control means.

Feature	Cohen’s *d*	Site $\eta^{2}$	Site *p* (KW)	Patient Group Mean Outside Control Site Mean Range
amplitude skewness	−0.72	0.095	0.777	Yes
hnr_mean	−1.07	0.181	0.611	Yes
shimmer_local	+0.79	0.254	0.214	Yes
shimmer_apq3	+0.66	0.250	0.227	No
chroma5_mean	−0.49	0.058	0.793	Yes
spectral_entropy	+0.73	0.079	0.735	No
f3_mean	−0.40	0.119	0.725	No
delta2_mfcc4_mean	+0.39	0.172	0.243	No
chroma6_mean	−0.34	0.160	0.300	No
f0_std	+0.43	0.105	0.313	Yes
f0_mean	−0.13	0.345	0.027	No
f2_mean	−0.06	0.248	0.133	No
mfcc6_mean	+0.43	0.292	0.029	No
jitter_local	+0.65	0.127	0.459	Yes
pitch_cv	+0.58	0.026	0.994	Yes

**Table 15:** Clinical correlation and medication analyses for the final Random Forest + DD15 + Method 2 (Original+Augmented/Original) model. Prediction-probability associations with clinical scores are Spearman correlations (ρ); correctly vs. misclassified comparisons use Mann–Whitney U tests, with a permutation test (10,000 iterations) for the PDI difference.

Analysis	Statistic	*p*
*Prediction probability vs. clinical scores*		
PANSS total (patients)	ρ=0.022	0.893
PANSS positive (patients)	ρ=0.206	0.195
PANSS negative (patients)	ρ=−0.102	0.527
CAPS total (controls)	ρ=−0.258	0.095
PDI total (controls)	ρ=−0.207	0.183
*Misclassification: correctly classified vs. misclassified*		
PANSS total (patients)	74.5±18.0 vs. 73.2±15.7	0.952
PANSS positive (patients)	15.5±5.2 vs. 14.4±3.5	0.738
PANSS negative (patients)	22.7±5.8 vs. 23.9±7.8	0.715
CAPS total (controls)	4.71±3.57 vs. 4.20±3.70	0.732
PDI total (controls; correct vs. false-positive)	4.26±2.14 vs. 1.60±1.34	0.011 * 0.009 ^†^
*Medication*		
CPZ dose (correct vs. incorrect patients)	–	0.681
Maximum change in PANSS partial correlations after CPZ adjustment	Δρ≤0.015	–

CPZ, chlorpromazine-equivalent dose. Misclassified controls are the five false positives; misclassified patients are the ten false negatives. * Uncorrected Mann–Whitney U comparison between the five false-positive controls and correctly classified controls. ^†^ Permutation test (10,000 iterations).

**Table 16:** Sex-stratified performance under Random Forest + DD15 + Method 2 (Original+Augmented/Original). AUC is reported as mean ± SD. Bold indicates the best value per metric.

Group	AUC	Acc.	Sens.	Spec.	F1 Score
Overall	0.887±0.078	0.823	0.753	0.889	0.799
Males	0.841±0.102	0.711	0.703	0.683	0.710
Females	** 0.923±0.106 **	**0.871**	**0.720**	**1.000**	**0.817**

## Data Availability

Participant data and study code are available upon request. Patient data are under the governance of the University Psychiatric Hospital “Vrapče”, Zagreb, Croatia, and healthy control data and code are under the governance of the Ericsson Nikola Tesla d.d., Zagreb, Croatia. Contact person: Luka Jelić (corresponding author).

## Supplementary Materials

The following supporting information can be downloaded at: https://www.mdpi.com/article/10.3390/s26196195/s1, Supplementary Section S1: Source–filter decomposition of amplitude skewness; Supplementary Section S2: Extended 50 feature set; Supplementary Section S3: Pretrained embedding and hybrid configuration comparison; Supplementary Section S4: Transfer to unprocessed full-length voice recordings; Supplementary Section S5: Additional recording-condition, preprocessing, and augmentation sensitivity analyses.

## Abbreviations

The following abbreviations are used in this manuscript:

AUC	Area under the receiver operating characteristic curve
APQ3	3-point amplitude perturbation quotient
ARI	Adjusted Rand index
AVH	Auditory verbal hallucinations
CAPS	Cardiff Anomalous Perceptions Scale
CI	Confidence interval
CPZ	Chlorpromazine equivalent
CV	Cross-validation
DC	Direct current
DD12	Data-driven 12-feature set
DD15	Data-driven 15-feature set
eGeMAPS	extended Geneva Minimalistic Acoustic Parameter Set
F1–F5	First through fifth voice formants
F1 score	Harmonic mean of precision and recall
HNR	Harmonic-to-noise ratio
ICD-10	International Classification of Diseases, 10th Revision
IQR	Interquartile range
KW	Kruskal–Wallis
LPC	Linear predictive coding
MFCC	Mel-frequency cepstral coefficients
PANSS	Positive and Negative Syndrome Scale
PDI	Peters et al. Delusion Inventory
PCA	Principal component analysis
RAP	Relative average perturbation
RBF	Radial basis function
RMS	Root Mean Square
ROC	Receiver operating characteristic
SD	Standard deviation
SHAP	SHapley Additive exPlanations
SNR	Signal-to-noise ratio
SVM	Support vector machine(s)
ZCR	Zero Crossing Rate
