# Supplementary material for: Acoustic Features of Sustained Phonation for Schizophrenia Classification: A Feasibility Study
Source: Sensors (Basel). 2026 Sep 30;26(19):6195. doi: 10.3390/s26196195 (PMC13667552; doi:10.3390/s26196195)
Supplement: Supplementary file 1 [file sensors-26-06195-s001.zip › sensors-4553952-supplementary.pdf]

Acoustic Features of Sustained Phonation for Schizophrenia Classification: A Feasibility Study - Supplementary Materials

Luka Jelić[1*,2], Kristian Jambrošić[1], Vinko Lešić[1], Pavo Orepić[3]

1 University of Zagreb Faculty of Electrical Engineering and Computing, 10000 Zagreb, Croatia, luka.jelic@fer.unizg.hr, kristian.jambrosic@fer.unizg.hr, vinko.lesic@fer.unizg.hr
2 Ericsson Nikola Tesla d.d., 10000 Zagreb, Croatia, luka.jelic@ericssonnikolatesla.com
3 University of Zurich, Linguistic Research Infrastructure, Department of Computational Linguistics, 8050 Zurich, Switzerland, pavo.orepic@uzh.ch
* Correspondence: luka.jelic@fer.unizg.hr

S1. Source–filter decomposition of amplitude skewness

S1.1 Introduction

Amplitude skewness was the most discriminative DD15 feature (Section 3.3). To better understand the acoustic information captured by this relatively uncommon feature, we examined its relationship with cycle-level waveform shape and established voice-quality measures. We then explored whether the *patient–control* difference was more consistent with contributions from the glottal source or the vocal-tract filter using LPC inverse filtering together with three complementary analyses: cepstral liftering, formant analysis, and spectral tilt.

Because the recordings had been peak-normalised, noise-reduced, and trimmed to a stable 500-ms segment as part of the parent study (Section 2.2), these analyses are exploratory and should not be interpreted as establishing a specific physiological mechanism.

S1.2 Methods

All analyses used the original, non-augmented 500-ms recordings from the 84 participants. To characterize the amplitude-skewness measure, whole-segment skewness was compared with mean skewness calculated across individual pitch cycles, and its relationships with HNR, shimmer, jitter, and spectral entropy were assessed using Pearson correlations.

To determine whether the group difference was more related to the glottal source or to the vocal tract, four complementary analyses were conducted:

- **LPC analysis:** the vocal-tract contribution was mathematically removed to estimate the underlying source signal, and we tested whether the *patient–control* skewness difference remained in this estimated source. A complementary

reconstruction then assessed whether this difference emerged when an artificial excitation signal was passed through each participant's estimated vocal-tract filter.

- **Cepstral analysis:** the signal was decomposed into components primarily associated with the slowly varying spectral envelope (vocal tract) and the harmonic structure (source), providing a complementary comparison of source- and filter-related contributions.
- **Formant analysis:** formant frequencies and bandwidths were examined as direct acoustic measures of vocal-tract resonances, with their associations with amplitude skewness also tested.
- **Spectral-tilt analysis:** H1-H2, an index reflecting glottal-source characteristics, was examined for its association with amplitude skewness.

Group differences were assessed using Mann-Whitney U tests and Cohen's *d*, calculated as *patient–control.* Pearson correlations were used to assess relationships between acoustic measures.

S1.3 Results

Waveform shape and relationships with other features. Whole-segment amplitude skewness was almost perfectly correlated with the average skewness of individual voice cycles ($r = 0.999$). The *patient−control* difference was also almost unchanged when skewness was calculated cycle by cycle ($d = -0.722$, $p = 0.025$). This indicates that the whole-segment measure largely reflected recurring waveform-shape differences rather than only slow variation across the 500-ms segment.

Amplitude skewness showed only weak correlations with HNR ($r = 0.104$, $p = 0.348$), local shimmer ($r = 0.074$, $p = 0.503$), APQ3 shimmer ($r = 0.104$, $p = 0.346$), and local jitter ($r = 0.008$, $p = 0.944$). It was moderately correlated with spectral entropy ($r = -0.454$, $p < 0.001$).

Source-filter analyses. Amplitude skewness was lower in patients than in controls ($d = -0.716$, $p = 0.027$). Across the complementary analyses, the overall pattern was more consistent with a vocal-tract/filter-related contribution than with the examined glottal-source measures (**Table S1**).

Table S1. Complementary exploratory analyses of amplitude skewness. Cohen's *d* is calculated as *patient – control*.

Analysis	Result	Interpretation
LPC source-filter analysis	Estimated source: d = −0.041, p = 0.775; vocal-tract reconstruction: d = 0.571, p = 0.044	The group difference was absent after estimating the source alone but appeared when the estimated vocal-tract filter was examined
Cepstral analysis	Source-related component: d = 0.122, p = 0.623; filter-related component: d = −0.885, p < 0.001	The larger group difference occurred in the component related to the vocal-tract filter
Formant analysis	F1 bandwidth: 181 vs.117 Hz, d = 0.758, p = 0.001; F2 bandwidth: 149 vs. 110 Hz, d = 0.469, p = 0.010; F1 frequency correlation with skewness (r = 0.343, p = 0.001)	Patients showed differences in vocal-tract resonance characteristics that were consistent with the source-filter findings
H1-H2	H1-H2 differed between groups (d = 0.271, p = 0.043) but was unrelated to skewness (r = 0.008, p = 0.940)	The skewness difference was not associated with this glottal-source-related measure

S1.4 Interpretation

Across the complementary analyses, the amplitude-skewness group difference was more consistent with a vocal-tract/filter-related contribution than with the examined glottal-source measures. Both LPC-based analysis and cepstral liftering showed substantially larger group differences in filter-related than source-related components, while the formant analyses provided additional evidence of vocal-tract-related differences. In contrast, amplitude skewness showed essentially no association with H1-H2 glottal-source index. The wider F1 and F2 bandwidths observed in patients are consistent with greater acoustic damping, although the underlying physiological or articulatory mechanism cannot be determined from these recordings.

Taken together, these findings suggest that amplitude skewness captured acoustic information that was largely distinct from conventional perturbation and harmonicity measures and that its *patient−control* difference was more closely associated with vocal-tract filtering than with the examined measures of glottal-source behaviour. The matched raw/processed analysis in Section S5.2.2 showed that amplitude skewness was strongly preserved in relative ordering but shifted systematically after preprocessing. Prospective

replication using standardized acquisition across balanced recording environments is still required before a mechanistic interpretation can be established.

S2. Extended 50 feature set

Table S2 lists the complete 50-feature pool described in Section 2.3, with acoustic-domain labels and indicators of membership in the DD15 and Literature 14 feature sets.

Table S2. A full list of Extended 50 features with domain tags. ✓ marks membership in DD15 (15 features) and Literature 14 (14 features).

#	Feature	Domain	DD15	L14
1	f0_mean	Prosodic	✓	✓
2	f0_std	Prosodic	✓	
3	pitch_cv	Prosodic	✓	
4	f1_mean	Formant		✓
5	f2_mean	Formant	✓	✓
6	f3_mean	Formant	✓	✓
7	f4_mean	Formant		✓
8	f5_mean	Formant		✓
9	jitter_local	Voice quality	✓	✓
10	jitter_rap	Voice quality		
11	shimmer_local	Voice quality	✓	✓
12	shimmer_apq3	Voice quality	✓	
13	hnr_mean	Voice quality	✓	✓
14	mfcc1_mean	Cepstral		✓
15	mfcc2_mean	Cepstral		✓
16	mfcc3_mean	Cepstral		✓
17	mfcc4_mean	Cepstral		✓
18	mfcc5_mean	Cepstral		✓
19	mfcc6_mean	Cepstral	✓	
20	mfcc7_mean	Cepstral		
21	mfcc8_mean	Cepstral		
22	mfcc9_mean	Cepstral		
23	mfcc10_mean	Cepstral		
24	mfcc11_mean	Cepstral		

#	Feature	Domain	DD15	L14
25	mfcc12_mean	Cepstral		
26	mfcc13_mean	Cepstral		
27	delta_mfcc1_mean	Cepstral		
28	delta_mfcc2_mean	Cepstral		
29	delta_mfcc3_mean	Cepstral		
30	delta_mfcc4_mean	Cepstral		
31	delta_mfcc5_mean	Cepstral		
32	delta_mfcc9_mean	Cepstral		
33	delta_mfcc10_mean	Cepstral		
34	delta_mfcc12_mean	Cepstral		
35	delta_mfcc13_mean	Cepstral		
36	delta2_mfcc4_mean	Cepstral	✓	
37	delta2_mfcc5_mean	Cepstral		
38	delta2_mfcc10_mean	Cepstral		
39	spectral_centroid_mean	Spectral		
40	spectral_bandwidth_mean	Spectral		
41	spectral_rolloff_mean	Spectral		
42	spectral_contrast_mean	Spectral		
43	spectral_flatness_mean	Spectral		
44	spectral_entropy	Spectral	✓	
45	chroma5_mean	Chroma	✓	
46	chroma6_mean	Chroma	✓	
47	chroma7_mean	Chroma		
48	zcr_mean	Time/energy		
49	rms_mean	Time/energy		
50	skewness	Distributional	✓	

S3. Pretrained embedding and hybrid configuration comparison

Pretrained wav2vec2-base and HuBERT-base embeddings were evaluated using Random Forest as the representative nonlinear classifier and logistic regression as a complementary linear model under both Method 1 (Original/Original) and Method 2 (Original+Augmented/Original) configurations. Random Forest was selected as the better performing of the two nonlinear models in the main analyses, while logistic regression provided a linear comparison. Hybrid DD15+embedding representations were evaluated

using Random Forest. For both pretrained models, “last” and “inter” refer to time-averaged embeddings from the final and fourth transformer layers. Embeddings were standardized and reduced using PCA before classification.

Table S3 reports cross-validated AUC for all pretrained-embedding and hybrid configurations evaluated under Method 1 (Original/Original) and Method 2 (Original+Augmented/Original), together with DD15 + Random Forest as the reference.

Table S3. Cross-validated AUC for pretrained embedding and hybrid configurations under Method 1 (Original/Original) and Method 2 (Original+Augmented/Original). Values are mean±SD across five folds. DD15 + Random Forest is included as the reference configuration.

Configuration	Method 1 (Original/Original)	Method 2 (Original+Augmented/Original)
DD15 + RF	**0.873±0.109**	**0.887±0.078**
wav2vec2 last + RF	0.637±0.109	0.706±0.141
wav2vec2 last + LR	0.760±0.085	0.681±0.102
wav2vec2 inter + RF	0.754±0.149	0.745±0.083
HuBERT last + RF	0.609±0.124	0.555±0.138
HuBERT last + LR	0.702±0.103	0.712±0.157
HuBERT inter + RF	0.814±0.126	0.833±0.137
Hybrid DD15 + wav2vec2 RF	0.841±0.113	0.862±0.103
Hybrid DD15 + HuBERT RF	0.863±0.094	0.862±0.104

The fixed DD15 values are included as a descriptive reference. Because DD15 was selected using the complete dataset, these comparisons should not be interpreted as evidence that DD15 outperformed the alternative representations. The fold-wise 15-feature estimate reported in the main manuscript (AUC = 0.837 ± 0.086) provides the more conservative internal estimate and is broadly comparable with the stronger alternative representations.

S4. Transfer to unprocessed full-length voice recordings

S4.1 Rationale

All voice recordings underwent peak normalization and background-noise removal as part of the parent study, followed by selection of a stable 500-ms segment (Section 2.2). These steps were used to provide more standardized stimuli for the perceptual task and to reduce differences in recording level and background noise across participants and recording locations. The same preprocessing procedure was applied to patients and controls.

Because preprocessing may nevertheless alter some acoustic properties, we additionally examined the original unprocessed recordings. We applied the final Random Forest classifier, trained on the standardized 500-ms segments, directly to the unprocessed full-

length recordings and examined whether amplitude skewness, the highest-ranked DD15 feature, also showed a patient-control difference in this input condition. Recording condition measures were additionally examined to characterize variation present before preprocessing.

S4.2 Methods

The final DD15 + Random Forest + Method 2 (Original+Augmented/Original) model was applied without retraining to features extracted from the unprocessed recordings at their original length. Of the 84 participants, three recordings could not be processed without manual repair, and two control recordings were unavailable, leaving 79 usable recordings (38 patients, 41 controls).

Group differences in loudness (RMS), absolute DC offset, and the proportion of spectral energy below 80 Hz were quantified using Cohen's *d*. The association between amplitude skewness and diagnosis was examined before and after accounting for RMS and low-frequency energy. This analysis represents a model transfer check from the standardized 500-ms input used in the main analysis to unprocessed full-length audio, rather than a re-derivation of DD15 using matched raw 500-ms segments.

S4.3 Results

Performance on unprocessed recordings. Direct application of the final model to the unprocessed full-length recordings resulted in an AUC of 0.639 (95% CI 0.514-0.763). At the default threshold, sensitivity was 0.974, whereas specificity was 0.024, indicating a strong shift toward patient-class predictions when the model was applied to this different input condition.

Large group differences were also observed in several recording-condition measures on the unprocessed recordings (**Table S4**).

Table S4. Group differences in recording-condition measures extracted from the unprocessed recordings (all $p < 1 \times 10^{-11}$). The large differences indicate substantial group-related variation in recording level and low-frequency signal characteristics in the original recordings.

Recording-condition variable	Patients	Controls	Cohen's *d*
Loudness (RMS)	0.094	0.019	2.24
Absolute DC offset	0.042	0.003	4.71
Low-frequency energy	0.00124	0.00013	1.12

Raw full-length skewness and recording conditions. Amplitude skewness remained associated with diagnosis in the unprocessed full-length recordings ($r = -0.345$, $p = 0.002$), indicating that a patient vs. control skewness difference was also detectable in this unprocessed full-length input condition. After accounting for RMS and low-frequency energy, the association between amplitude skewness and diagnosis decreased (r =

−0.042, p = 0.71), suggesting that recording conditions may have contributed to the observed association in the raw audio recordings.

S4.4 Interpretation

The preprocessing used in the main analysis was applied identically to patients and controls and was intended to provide more standardized stimuli for the parent study voice perception task, particularly given differences in recording location, voice level, and background noise. However, direct application of the model trained on these standardized 500-ms segments to the original unprocessed full-length recordings resulted in substantially lower performance, particularly in specificity. This suggests that the classifier relies on consistent input and a consistent preprocessing pipeline and did not generalize well to these substantially different input conditions.

From a practical perspective, this also suggests that some form of input standardization may be important for future clinical applications, where recording conditions may not be fully controlled. Models trained on standardized recordings should not be expected to generalize directly to substantially different raw inputs without the appropriate preprocessing or retraining.

The raw recordings revealed significant differences in RMS, absolute DC offset and low-frequency energy between patients and controls, confirming substantial variation in recording conditions prior to preprocessing. Amplitude skewness was also associated with diagnosis in the unprocessed full-length recordings. Because both preprocessing and segment duration differed between the standardized and unprocessed input conditions, this should be interpreted as evidence that a patient–control difference was detectable under both conditions rather than as a matched demonstration of robustness to preprocessing.

Accounting for RMS and low-frequency energy reduced the skewness-diagnosis relationship in the raw recordings, suggesting that its magnitude was sensitive to recording conditions. This limits the direct interpretation and transferability of amplitude skewness in unprocessed audio. The group difference observed under both input conditions nevertheless supports further investigation of this feature, but does not establish its robustness to preprocessing or recording environment. Together with its relatively small recording-site effect in the main analysis ($\eta^2 = 0.095$, Table 14) and the complementary source-filter findings in Supplementary Section S1, these results support further investigation of amplitude skewness as a potentially informative acoustic feature in schizophrenia. A matched same-window analysis addressing preprocessing more directly is reported in Section S5.2.2.

S5. Additional recording-condition, preprocessing, and augmentation sensitivity analyses

S5.1 Rationale and Methods

The full-length transfer analysis in Section S4 intentionally compared substantially different input conditions and therefore could not isolate the effects of preprocessing from those of segment duration. To address the remaining recording condition and preprocessing questions more directly, additional sensitivity analyses were performed on the same processed 500-ms inputs used in the main classification analysis and, where possible, on their corresponding raw source intervals.

S5.1.1. Processed 500-ms recording condition analyses

RMS, absolute DC offset, and the proportion of spectral energy below 80 Hz were calculated for the processed 500-ms segment from each of the 84 participants. Patient−control differences were assessed using two-sided Mann−Whitney U tests and Cohen's d (patient minus control). The association between processed segment amplitude skewness and diagnosis was examined before adjustment and after adjustment for RMS plus low-frequency energy, and for RMS plus absolute DC offset plus low-frequency energy, by residualizing both skewness and diagnosis on the recording condition variables. Because these variables were strongly right-skewed, the sensitivity analyses were repeated after log10 transformation of low-frequency energy and, where included, absolute DC offset. Bootstrap 95% confidence intervals were obtained from 10,000 resamples in which the adjustment models were refitted within each resample.

S5.1.2. Classifier adjustment

To examine whether the measured recording condition variables contributed to DD15 classification, Random Forest Method 1 (Original/Original) was repeated on the same five participant-level folds. Within each fold, each DD15 feature was residualized on RMS, absolute DC offset, and low-frequency energy using coefficients estimated from the training participants only. These coefficients were then applied to the held-out participants before classification. Method 1 was used so that recording condition measures were directly defined for every training and test recording, avoiding assignment of recording condition values from the original recordings to augmented copies.

S5.1.3. Matched raw-versus-processed analysis

For each participant with an available raw recording, the processed 500-ms segment was matched back to its temporal source interval in the unprocessed full-length recording. Matching used sliding normalized cross-correlation on the waveform and on an 80−5000 Hz band-passed version, followed by refinement at the raw file's native sampling rate. Matches were accepted only when the combined correlation was at least 0.85, the best match exceeded a distant alternative by at least 0.03, and the full-band and band-passed

lags agreed within 20 ms. The same feature extraction implementation used in the main analysis was then applied to the matched raw and processed segments for amplitude skewness, local jitter, local shimmer, APQ3 shimmer, and HNR.

S5.1.4. Augmentation analysis

For each of the 84 participants, the five augmented copies were compared with that participant's original processed 500-ms segment for the same five acoustic measures. Since the five observations from each participant were not independent, the augmentation-induced changes were averaged within each participant before inferential testing. Original-to-augmented correlations, signed changes, absolute changes standardized by the original feature distribution, and paired Wilcoxon tests were then summarized at the participant level.

S5.2 Results

S5.2.1. Processed 500-ms recording-condition analyses and adjustment analyses

The very large raw-recording RMS difference reported in Section S4 was substantially reduced after preprocessing, with no clear patient−control difference remaining in processed RMS. Residual group differences remained in absolute DC offset and low-frequency energy, although their standardized effect sizes were much smaller than in the unprocessed full-length recordings (**Table S5**).

Table S5. Group differences in recording-condition measures extracted from the processed 500-ms segments. Values are mean ± SD. p-values are from two-sided Mann−Whitney U tests; Cohen's d is calculated as patient − control.

Measure	Patients	Controls	Cohen's d	p-value
RMS	0.056343 ± 0.052333	0.066201 ± 0.056341	−0.181	0.168
Absolute DC offset	0.000298 ± 0.000602	0.000060 ± 0.000046	0.566	1.9×10^{-8}
Low-frequency energy <80 Hz	0.000246 ± 0.000460	0.000049 ± 0.000041	0.609	2.4×10^{-4}

Amplitude skewness remained associated with diagnosis in the processed segments before adjustment (r = −0.343, p = 0.0014). Using the same untransformed recording condition variables as in Section S4, weaker residual associations remained after adjustment. However, when the strongly skewed recording condition variables were log-transformed, the adjusted associations were smaller, with their bootstrap confidence intervals covering zero (**Table S6**the specification of the recording condition variables.

Table S6). Therefore, the adjusted skewness-diagnosis association was sensitive to the specification of the recording condition variables.

Table S6. Association between processed-segment amplitude skewness and diagnosis before and after adjustment for measured recording-condition variables. Adjusted-model confidence intervals are bootstrap 95% CIs.

Adjustment	r	p-value	95% CI
Unadjusted	−0.343	0.0014	—
RMS + low-frequency energy	−0.227	0.040	[−0.405, −0.008]
RMS + absolute DC offset + low-frequency energy	−0.248	0.025	[−0.455, −0.044]
RMS + log10(low-frequency energy)	−0.121	0.280	[−0.309, 0.068]
RMS + log10(absolute DC offset) + log10(low-frequency energy)	−0.153	0.172	[−0.337, 0.024]

Using the same five participant-level folds, the unadjusted DD15 + Random Forest Method 1 analysis reproduced the manuscript result (AUC = 0.873 ± 0.109). After leakage-safe within-fold residualization of DD15 features on RMS, absolute DC offset, and low-frequency energy, mean AUC decreased to 0.806 ± 0.089 (Δ = −0.067). This suggests that the measured recording condition variables contributed to the classification performance, but adjusting for these three measured proxies did not eliminate the internal discrimination. Still, these measures are proxies for recording conditions and cannot account for unmeasured or nonlinear patient-site effects.

S5.2.2. Matched raw-versus-processed 500-ms analysis

Of the 79 participants with available raw recordings, 64 produced high confidence temporal matches (31 patients and 33 controls). Matching success was similar in patients (31/38; 81.6%) and controls (33/41; 80.5%; Fisher exact p = 1.00). The accepted matches had a median refined native rate correlation of 0.998 (range 0.980−1.000). There were no clear overall differences between participants with and without accepted matches across the examined acoustic and recording condition measures. However, an isolated difference in absolute DC offset was observed within the control group. The matched comparison is therefore treated as a sensitivity analysis rather than a complete cohort preprocessing ablation.

Jitter, HNR, and amplitude skewness were strongly preserved in relative ordering between the matched raw and processed segments, whereas shimmer measures showed more moderate correspondence (**Table S7**). HNR decreased by 0.221 dB on average after preprocessing.

Table S7. Correspondence between acoustic measures extracted from high-confidence matched raw and processed 500-ms segments (n = 64).

Feature	Pearson r	Spearman ρ	Observed preprocessing effect
Amplitude skewness	0.986	0.969	Strong relative preservation; systematic absolute shift
Jitter local	0.993	0.985	Very strong preservation; small average shift
Shimmer local	0.735	0.768	Moderate preservation; more processing-sensitive
Shimmer APQ3	0.725	0.768	Moderate preservation; more processing-sensitive
HNR mean	0.995	0.993	Strong relative preservation; mean change −0.221 dB

Several patient−control differences were already detectable in the matched raw recording segments: amplitude skewness ($d = -0.841$, $p = 0.029$), local jitter ($d = 0.600$, $p = 0.012$), local shimmer ($d = 0.405$, $p = 0.019$), and HNR ($d = -1.042$, $p < 0.001$). Raw shimmer APQ3 did not show a clear group difference ($d = 0.219$, $p = 0.132$). These results indicate that the corresponding group differences were not created purely by preprocessing, although their magnitude may have changed because of processing.

The preprocessing induced HNR change was larger in patients than controls (mean processed-minus-raw change −0.360 vs. −0.089 dB; $d = -0.788$, $p = 0.00037$). Group comparisons of preprocessing induced change were not statistically clear for skewness, jitter, local shimmer, or APQ3 (all $p > 0.32$). Therefore, processed values, particularly those relating to HNR and shimmer, should not be assumed to be numerically interchangeable with values obtained from the raw waveform.

S5.2.3. Effect of augmentation on acoustic measures

Augmentation preserved participant ordering relatively well for amplitude skewness and jitter but produced larger changes in shimmer and HNR (**Table S8**). The synthetic copies should therefore be treated as training perturbations rather than acoustically equivalent repeated recordings.

Table S8. Effect of augmentation on selected acoustic measures. Correlations compare each original recording with the participant mean across five augmented copies. Mean |standardized change| is the mean absolute change divided by the SD of the original feature.

Feature	Pearson r	Spearman ρ	Mean signed change	Mean |standardized change|	Wilcoxon p
Amplitude skewness	0.983	0.981	+0.0222	0.126 SD	0.0185
Jitter local	0.912	0.955	−0.000607	0.414 SD	1.1×10^{-13}
Shimmer local	0.707	0.738	+0.0166	0.813 SD	5.7×10^{-13}
Shimmer APQ3	0.793	0.775	+0.00547	0.547 SD	1.1×10^{-10}
HNR mean	0.794	0.775	−3.053 dB	0.831 SD	2.0×10^{-15}

These feature-level changes qualify the interpretation of the small Method 1-to-Method 2 difference for DD15 + Random Forest (0.873 ± 0.109 vs. 0.887 ± 0.078). The numerical increase is small relative to fold-to-fold variability and is not interpreted as definitive evidence that augmentation improved the pipeline. Method 2 remains the final training configuration used for the DD15 analyses, with evaluation performed exclusively on original held-out recordings.

S5.3 Interpretation

The additional sensitivity analyses refine, rather than replace, the conclusions of the original full-length transfer analysis in Section S4. Preprocessing substantially reduced the very large raw RMS difference, but measurable group differences in absolute DC offset and low-frequency energy remained in the processed 500-ms inputs. The processed segment skewness association was attenuated after adjustment for these variables and was sensitive to recording condition variable specification. Likewise, within-fold adjustment of DD15 reduced Random Forest AUC from 0.873 to 0.806, indicating that the measured recording condition variables contributed to classifier discrimination. At the same time, adjustment for these measured proxies did not eliminate internal discrimination.

The matched analysis also showed that several patient−control acoustic differences were already present in the corresponding unprocessed source segments, while demonstrating that preprocessing can change the magnitude of particular measures, especially shimmer

and HNR. The augmentation analysis similarly showed that the synthetic perturbations are not acoustically neutral with respect to micro-acoustic measures.

These findings support treating the identified features as candidate signals for prospective replication rather than as processing-invariant biomarkers and treating augmentation as a training perturbation rather than simulated within-participant variability. Because diagnosis remains structurally confounded with the patient recording environment, balanced multi-site validation in which both diagnostic groups are represented across recording environments remains necessary.
